# Single-cell RNA-sequencing of stria vascularis cells in the adult *Slc26a4*^-/-^ mouse

**DOI:** 10.1186/s12920-023-01549-0

**Published:** 2023-06-15

**Authors:** Jin-Young Koh, Corentin Affortit, Paul T. Ranum, Cody West, William D. Walls, Hidekane Yoshimura, Jian Q. Shao, Brian Mostaert, Richard J.H. Smith

**Affiliations:** 1 Roy J. Carver Department of Biomedical Engineering, College of Engineering, University of Iowa, University of Iowa, Iowa City, IA USA; 2 Molecular Otolaryngology and Renal Research Laboratories, Carver College of Medicine, University of Iowa, Iowa City, IA USA; 3 Raymond G. Perelman Center for Cellular and Molecular Therapeutics, The Children’s Hospital of Philadelphia Research Institute, Philadelphia, PA USA; 4 Department of Otorhinolaryngology - Head and Neck Surgery, Shinshu University School of Medicine, Matsumoto, Japan; 5 Central Microscopy Research Facility, University of Iowa, Iowa City, IA USA; 6 Department of Otolaryngology, Head and Neck Surgery, Carver College of Medicine, University of Iowa, Iowa City, IA USA

**Keywords:** Single-cell RNA sequencing, Stria vascularis cell, Spindle cells, Hearing loss, *Slc26a4*, Pendred syndrome, DFNB4, Annexin A1

## Abstract

**Background:**

The primary pathological alterations of Pendred syndrome are endolymphatic pH acidification and luminal enlargement of the inner ear. However, the molecular contributions of specific cell types remain poorly characterized. Therefore, we aimed to identify pH regulators in pendrin-expressing cells that may contribute to the homeostasis of endolymph pH and define the cellular pathogenic mechanisms that contribute to the dysregulation of cochlear endolymph pH in *Slc26a4*^−/−^ mice.

**Methods:**

We used single-cell RNA sequencing to identify both *Slc26a4*-expressing cells and *Kcnj10*-expressing cells in wild-type (WT, *Slc26a4*^+/+^) and *Slc26a4*^−/−^ mice. Bioinformatic analysis of expression data confirmed marker genes defining the different cell types of the stria vascularis. In addition, specific findings were confirmed at the protein level by immunofluorescence.

**Results:**

We found that spindle cells, which express pendrin, contain extrinsic cellular components, a factor that enables cell-to-cell communication. In addition, the gene expression profile informed the pH of the spindle cells. Compared to WT, the transcriptional profiles in *Slc26a4*^−/−^ mice showed downregulation of extracellular exosome-related genes in spindle cells. Immunofluorescence studies in spindle cells of *Slc26a4*^−/−^ mice validated the increased expression of the exosome-related protein, annexin A1, and the clathrin-mediated endocytosis-related protein, adaptor protein 2.

**Conclusion:**

Overall, cell isolation of stria vascularis from WT and *Slc26a4*^−/−^ samples combined with cell type-specific transcriptomic analyses revealed pH-dependent alternations in spindle cells and intermediate cells, inspiring further studies into the dysfunctional role of stria vascularis cells in *SLC26A4*-related hearing loss.

**Supplementary Information:**

The online version contains supplementary material available at 10.1186/s12920-023-01549-0.

## Background

Pendred syndrome (PDS, OMIM #274,600) and nonsyndromic enlarged vestibular aqueduct (NSEVA/DFNB4, OMIM #600,791) are both associated with variants in *SLC26A4* [1, 2]. The former is the second most common type of autosomal recessive syndromic deafness, accounting for approximately 7.5% of genetic hearing loss in humans [3], while the latter is the most common radiological malformation associated with childhood sensorineural hearing loss (SNHL) [4]. PDS also manifests with euthyroid goiter in the thyroid gland [1, 2]. Although digenic inheritance is very rare (< 1%), the PDS and NSEVA/DFNB4 disease spectrum has also been reported as a consequence of compound heterozygosity of variants in *SLC26A4* with variants in *KCNJ10*, *FOXI1*, or *EPHA2* [5–7]. The hearing loss phenotype of the PDS and NSEVA/DFNB4 patient is fluctuating and progressive [1, 8, 9].

The *SLC26A4* gene encodes pendrin, an anion exchanger (such as I^−^, Cl^−^, and HCO_3_^−^) [10, 11] expressed in several tissues, including the thymus, kidney, and inner ear (cochlea, vestibular system, and endolymphatic sac [12]). In the inner ear, pendrin is expressed in the epithelial cells, transports bicarbonate (HCO_3_^−^) into the endolymph, and maintains endolymphatic pH homeostasis. Lack of pendrin in *Slc26a4*^−/−^ mice results in hearing loss, vestibular dysfunction, and abnormal morphology of the bony labyrinth including an enlarged vestibular aqueduct and a Mondini-like dysplasia (i.e., abnormal cochlear turn) [13]. Previous studies have shown that pendrin maintains endocochlear potential (EP) and endolymph pH, which are essential for endolymph homeostasis and normal auditory function [11, 14–16]. The underlying cellular and molecular mechanisms required to maintain EP and endolymph pH are largely unknown.

In general, endolymph pH is finely regulated by H^+^, HCO_3_^−^ [17], and carbonic anhydrases (CAs) [18]. In the cochlea, where the pH is about 7.5 [19], H^+^-ATPase is highly expressed in several endolymph-facing cells [20], but it is the Na^+^/H^+^ exchangers (NHEs), encoded by *Slc9a3*, that are known to transport H^+^ ions into the endolymph [21]. During inner ear development, cytosolic isozymes (*Car1*, *Car2*, *Car3*, and *Car13*) and membrane-bound isozymes (*Car12* and *Car14*) are more highly expressed than other isozymes of the carbonic anhydrase gene family [22]. However, cell-specific differences in CA expression are not known.

In the *Slc26a4*-insufficiency mouse model, degeneration of *Kcnj10*-expressing (intermediate) cells in the cochlear stria vascularis (SV) is observed in association with fluctuating hearing loss; no other abnormalities are detected in the cochlea [23]. Additionally, the early (P6) re-induction of *Slc26a4* reduces the fluctuation of hearing and increases pendrin expression in spindle cells of the SV [24]. These findings suggest that gene expression profiling at the single-cell level in cochlear SV, including intermediate and spindle cells, may provide further insight into the pathophysiology of PDS and NSEVA/DFNB4.

With that goal in mind, in this work we used single-cell RNA-sequencing (scRNA-seq) to define SV cell types and factors in regulating cochlear endolymph pH. We sought to identify changes in *Slc26a4*^−/−^ mice to clarify cellular and molecular mechanisms underlying *SLC26A4*-related hearing loss (Fig. S1). We found pH regulators in cochlear spindle cells (SCs) that may aid in maintaining endolymph homeostasis. In addition, we identified genes showing transcriptional perturbation in mutant animals when endolymph pH is reduced, thus establishing putative underlying cellular and molecular mechanisms of PDS and NSEVA/DFNB4.

## Methods

### Sample preparation

#### Animals

All mice were kept under temperature-controlled (22-23 °C), light-controlled (12-hour light cycle from 6 AM to 6 PM), and humidity-controlled (40-60%) conditions with free access to food and water. The Iowa Institutional Animal Care and Use Committee approved all work. Animals were euthanized before cochlear dissection and single-cell isolation according to American Veterinary Medical Association guidelines [25]. For scRNA-seq experiments, *Slc26a4*^+/−^ (n = 10), *Slc26a4*^−/−^ (n = 21), and *Slc26a4*^+/+^ mice (n = 55) on 129S6 (Taconic Biosciences) background were used. All mice were male and of postnatal (P) age (22–42 days).

#### Single-cell isolation

We isolated single cells from the murine cochlea following NIH guidelines for the care and use of laboratory animals [26, 27]. After euthanasia by CO_2_, the temporal bones were removed and placed into Dulbecco’s phosphate-buffered saline (DPBS, 14190144 Gibco). Here, we removed and opened the temporal bone under a dissecting microscope (M165FC; Leica Microsystems) to access the cochlear membranous labyrinth. Once the encapsulating bone was removed, we carefully removed the stria vascularis tissue from the lateral wall of the spiral ligament and incubated it for 5 min at 37 °C with collagenase from clostridium histolyticum (C5138; Sigma Aldrich) in solution at a working concentration of 0.3 mg/500µl. Cell dissociation was completed by gentle trituration. The resulting suspension was placed under an inverted microscope (DMI3000B; Leica Microsystems) equipped with 20x and 40x objectives and two 3D micromanipulators (MN-153; Narishige), each driving a pulled-glass micropipette attached to a nitrogen gas-powered Pico-Injector (PLI-100; Harvard Apparatus) to control aspiration pressure. The dissociated cells were examined under the inverted microscope and were aspirated into the first pulled-glass micropipette (FG-GBF150-86-10; Sutter Instrument) in a slow and controlled manner and then washed in fresh DPBS to remove residual collagenase solution and debris.

#### Full-length cDNA preparation

Each washed single-cell was immediately re-aspirated using a second clean pulled-glass micropipette and expelled into a 0.2-ml thin-walled PCR tube containing lysis buffer, 0.2% Triton X-100 (T9284; Sigma-Aldrich), 10 µM oligo-dT primer, 10 mM dNTP mix (18427013; Invitrogen), and 2 U µl^− 1^ RNase inhibitor (10777019 Invitrogen) [27, 28] (within under 30 min, if possible). Lysed cells were stored on ice in an individual 0.2-ml thin-walled PCR tube until the ongoing batch of isolations was completed.

Reverse transcription and PCR amplification were performed sequentially to generate complementary DNA (cDNA) libraries using the SMART-Seq2 protocol and Superscript III Reverse Transcriptase (18080093; Invitrogen). In addition, the number of PCR amplification cycles was increased from 22 to 25 to improve yields. After cDNA purification using Ampure XP beads (17022200 Beckman Coulter), cDNA quality was checked using the Agilent 2100 Bioanalyzer and high-sensitivity DNA chip kits (5067 − 4626; Agilent Technologies, Santa Clara, CA). As a result, all full-length cDNAs peaked at ~ 1.5–2 kb [27, 28].

### Library preparation and single-cell RNA-sequencing (scRNA-seq)

For Illumina sequencing library preparation, Nextera XT DNA sample preparation kits (15032354; Illumina), including Tn5 enzymes for tagmentation, were used together with Nextera XT index kits (15055294; Illumina) for multiplexing. Tagmentation and amplification of adaptor-ligated fragments were carried out using half-volume reactions. For tagmentation, the input DNA was 0.125 ng. The number of amplification cycles for adaptor-ligated fragments was 14. After the adaptor-ligated single-cell cDNA libraries were purified, single-cell cDNA libraries were run on the Agilent 2100 Bioanalyzer high-sensitivity DNA chips and combined in equimolar concentrations based on index balance. We ran four batches containing n = 69, 73, 75, and 81 cells, respectively (batch1: 69 cells, 120 nM, 444 µl; batch2: 73 cells, 30 nM, 393.61 µl; batch3: 75 cells, 25 nM, 185.27 µl; batch4: 81 cells, 54 nM, 284.33 µl). The concentration of pools was measured with the Qubit dsDNA HS Assay Kit (Q32854; Invitrogen) and Qubit 2.0 Fluorometer (Q32866; Invitrogen). Before sequencing, free primers were removed with AMPure XP beads (x2) (A63881; Beckman Coulter).

Pooled single-cell cDNA libraries were sequenced on a single lane of Illumina HiSeq 4000 systems with 150-bp Paired-End reads at the Genomics Division in the Iowa Institute of Human Genetics (IIHG).

### Bioinformatic analysis

#### scRNA-seq dataset of Illumina sequencing

After Illumina sequencing, raw sequences were exported, and fastq files were aligned to the mm10 transcriptome (GRCm38) with an index built using the Kallisto pseudo-aligner [29]. Next, normalized estimated counts and transcripts per kilobase million (TPM) were quantified for each cell’s transcript (n = 298) and converted to a genes per cell counts matrix file using a customized Python script.

The genes per counts matrix file was input into Seurat [30], an R package for single-cell analysis. To determine the quality of the scRNA-seq data, we ran the standard pre-processing steps in the Seurat, including quality control metrics, data normalization, scaling, and detecting highly variable features. We identified 2,000 highly variable genes to employ a dimensional reduction technique, a principal component analysis (PCA). We then calculated the linear dimensional reduction on the scaled data. The optimal principal components (PCs 1–4) were determined by examining both a strong enrichment of low p-value features and the percentage of cell (n = 172) variance in WT mice (n = 55).

#### Unbiased clustering of cells

The data for qualified cells underwent a global-scaling normalization method that normalized the gene expression measurements for individual cells by total expression. Data were then multiplied by a scale factor (10,000) to log transform the results. In this process, we identified 2,000 genes showing variable expression profiles. Prior to PCA, we performed a linear transformation (‘scaling’) using the ScaleData function in the Seurat R package. This function shifts the expression of each gene so the mean expression across the cell is zero. In addition, the expression of each gene was adjusted so that the variance across cells was 1. This step scaled downstream analysis so that highly expressed genes would not dominate.

**Fig. 1:**
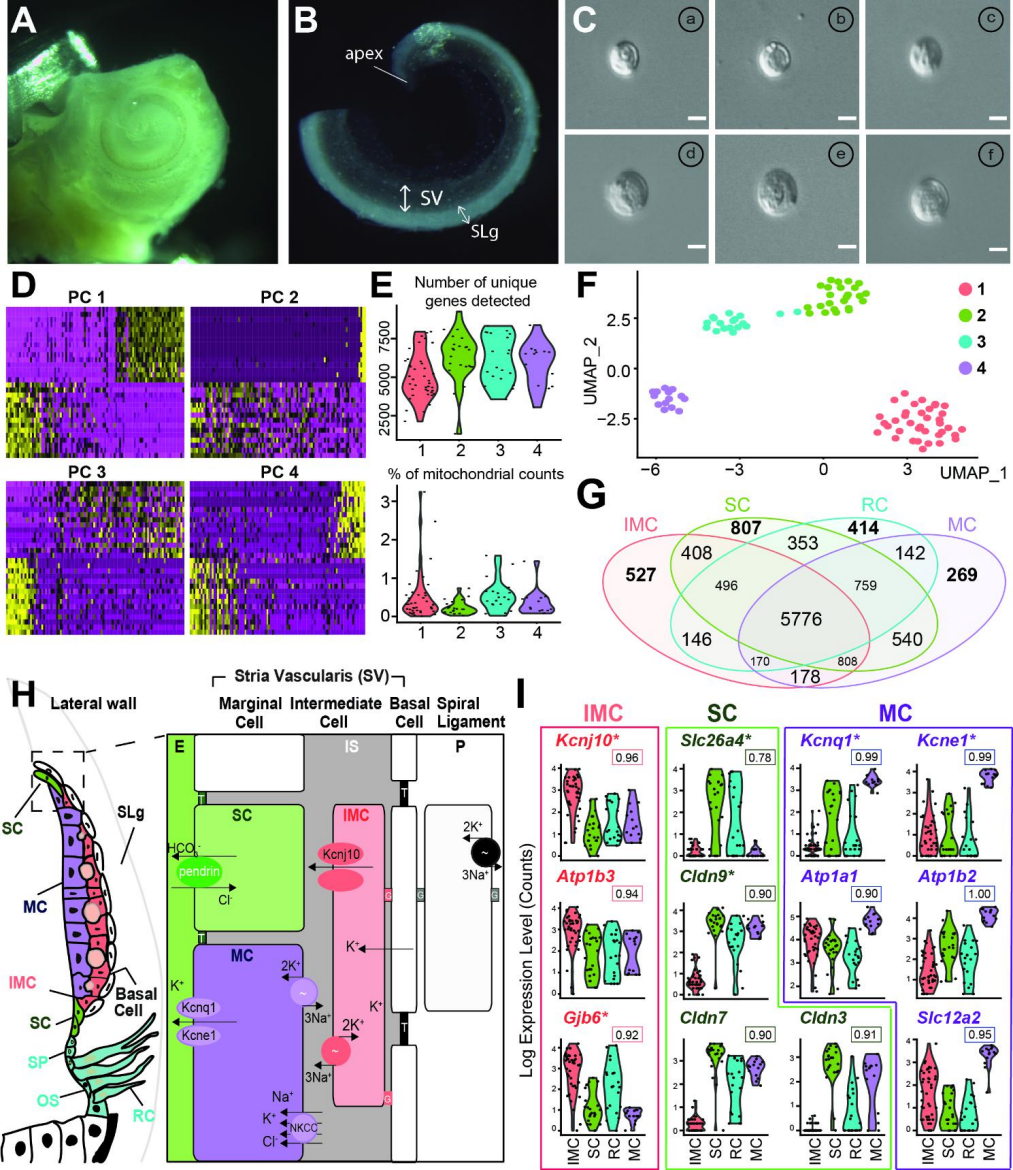
Single-cell isolation and unbiased clustering of the cochlear stria vascularis. **A-B**: The image shows mouse cochlea (A) and isolated stria vascularis (B). The bony labyrinth is opened at the apex to access the membranous labyrinth (SV, stria vascularis; SLg, spiral ligament). **C**: Representative picture of isolated single cells. Note: the varied morphology. (a), (b) and (c) are *Kcnj10*-expressing cells. (d), (e) and (f) are *Slc26a4*-expressing cells. Scale bar: 10 μm. **D**: Principal component analysis (PCA). Heatmaps of PCs 1–4 show differentially expressed genes. PCs 1–4 were later used for unbiased clustering. X-axis, cells; Y-axis, genes ordered by PCA score. **E**: Violin plot showing the number of unique genes detected per cell (top), and the percentage of mitochondrial counts (bottom) (group 1, pink; 2, green; 3, cyan; 4, purple). Each dot represents an individual cell. **F**: UMAP clustering displays four distinct clusters. **G**: Venn diagram showing the number of shared genes unique to each cell type. **H**: Schematic representation of the ion transporters and junctions in the cochlear SV. The marginal cell layer (spindle and marginal cells) expresses tight junctions (*Cldn9*, *Cldn7*, *Cldn3*). Between the marginal and basal cell layers is the intermediate cell (IMC) layer with K^+^ channels (*Kcnj10*), Na^+^/K^+^-ATPase (*Atp1b3*), and gap junctions (*Gjb6*). The marginal cell (MC) transports K^+^ into endolymph (E) using K^+^ channels (*Kcne1*/*Kcnq1*), Na^+^/K^+^-ATPase (*Atp1a1* and *Atp1b2*), and NKCC1 co-transporters (*Slc12a2*). The spindle cell (SC) expresses pendrin (*Slc26a4*) to maintain endolymph pH (G, gap junctions; OS, outer sulcus; RC, root cell; SLg, spiral ligament; SP, spiral prominence; T, tight junctions). **I**: Violin plots of ion transporters and junctions shown in (**H**). The ROC AUC score of each gene is indicated under the name of the gene. (*, deafness-causing genes, the complete list of deafness-causing genes is available in supplementary table S1)

A K-nearest neighbor (KNN) graph was constructed based on the Euclidean distance in PCA space using PCs 1–4. The KNN graph divided highly interconnected ‘quasi-cliques’ or ‘communities’ with edges drawn between cells with similar feature expression patterns. The KNN graph used nodes (n = 172) to refine the edge weights between any two cells based on the shared overlap in their local neighborhoods. We optimized the modularity through the Louvain algorithm. To determine how many PCs were needed, we visualized PCs using the JackStrawPlot function (Fig. S3A), ElbowPlot function (Fig. S3B), and the DimHeatmap function (Fig. 1D) in the Seurat R package.

We performed nonlinear dimensional reduction techniques to visualize the dataset (uniform manifold approximation and projection (UMAP)) [31]. To find differentially expressed genes of each cluster, we used a receiver operating characteristic (ROC) curve classifier for each group compared to all other clusters. The ROC curve was plotted by the true positive rate (sensitivity) against the false positive rate (1 - specificity). The area under a classifier’s ROC curve (AUC) was comparable to the probability that the classifier ranks a randomly selected positive instance higher than a randomly chosen negative instance. To visualize the cluster-defining genes in each group, genes were ordered by adjusted p-value based on Bonferroni correction using all genes in the dataset. Then, genes were ranked by log fold-change of the average expression among the groups.

#### Gene set enrichment analysis

To gain insights into the cellular components of SV cells, we leveraged an analytical method, Gene Set Enrichment Analysis (GSEA) [32]. To find Gene Ontology (GO) terms (Cellular Component, Molecular Function, Biological Process), we used the PANTHER classification system [33]. Fisher’s Exact test was used, and the correction was done by calculating the false discovery rate (FDR).

#### Cell type filtering

Of the total number of sequenced cells (n = 298) in four batches, 281 were SV cells, as a small number of auditory hair cells (n = 17) was included to check the sequencing quality in the first batch. Of these 281 cells, 172 cells were obtained from *Slc26a4*^+/+^ mice (n = 55), 40 from *Slc26a4*^−/−^ mice (n = 10), and 69 from *Slc26a4*^−/−^ mice (n = 21).

172 cells from *Slc26a4*^+/+^ mice (n = 55) were used for cell type identification. After unbiased cell clustering, we examined the total number of integrated SV cells (n = 281) to filter based on gene expression levels of cluster-defining genes. On this basis, 36 cells with low expression levels of cluster-defining genes were excluded. The total number of integrated SV cells, including cells from mutant mice, was 245.

### Differential expression analysis between genotypes

Of these 245 cells, 138 cells were obtained from WT mice (n = 55), 38 cells from *Slc26a4*^−/−^ mice (n = 10), and 69 cells from *Slc26a4*^-/-^ mice (n = 21). Differentially expressed (DE) genes were identified in intermediate cells (IMCs) and pendrin cells (PDCs) (spindle cells (SCs) and root cells (RCs)) using single-cell differential expression (SCDE) analysis [34]. The filtered number of SV cells (n = 245) were categorized by cluster-defining genes as IMCs, n = 131(WT n = 69, *Slc26a4*^−/−^ n = 23, and *Slc26a4*^-/-^ n = 39 cells, respectively) and PDCs, n = 89 (WT n = 51, *Slc26a4*^−/−^ n = 13, and *Slc26a4*^-/-^ n = 25 cells, respectively). We compared genotypes within the same cell type after checking cluster-defining genes in each cell type using Seurat and manual filtering because cell difference was more significant than genotype difference. The estimated counts observed for each gene were modeled using a mixture of a negative binomial (NB) distribution (for the detected transcripts) and low-level Poisson distribution (for the background-level signal of genes that were not detected) [35]. These models were then used to identify strongly differentially expressed genes between groups of cells. After fitting error models, poor cells displayed abnormal fit (most commonly showing negative correlation) and were removed. To test for differential expression between groups of cells, 2-tailed p-values were adjusted to control for FDR; genes with FDR < 0.05 were considered significant. The log2 fold expression difference value was reported as a maximum likelihood estimate (mle), with 95% lower bound (lb) and upper bound (ub) of the log2 fold expression difference value, and as a p-value.

### Tissue section preparation for immunofluorescence staining

After euthanasia, the cochlea and kidney were quickly fixed in 4% paraformaldehyde (15710; Electron Microscopy Sciences) for 1 h. Kidney tissues were submerged sequentially in 10%, 20%, and 30% sucrose. Next, tissues were put in the tissue-tek optimum cutting temperature (OCT) compound (4583; Sakura Finetek) and immediately frozen in liquid nitrogen. Sections were prepared using a Leica Microtome RM2135 (Leica, Germany). Cochlea tissues were decalcified for 48 h and then cryopreserved by progressive incubation from a solution of 20% sucrose to pure OCT. Tissues were embedded in fresh OCT and stored at -80 °C before sectioning using a Leica Microtome RM2135 (Leica, Germany).

### Immunofluorescence staining

Frozen cochlear sections were blocked and permeabilized with a 30% normal donkey serum solution and 0.3% Triton X-100 in 1X PBS. Immunocytochemistry was performed in cochlear transversal cryostat sections from WT and *Slc26a4*^−/−^ mice aged one month. Primary antibodies were incubated overnight in 1% normal donkey serum solution and 0.1% Triton X-100 in 1X PBS. Cochlear samples were immunostained with anti-AP2mu (1:150, Thermo Fisher Scientific Cat# MA5-35066, RRID: AB_2848971), anti-KCNJ10 (1:150, Alomone Labs Cat# APC-035, RRID: AB_2040120), anti-ANXA1-488 (1:250, Abcam, Cat#ab225513), anti-carbonic anhydrase 13 primary rabbit antibody (1:100, Proteintech Cat# 16696-1-AP, RRID: AB_1850972), and anti-Pendrin (1:100, #2842). Alexa Fluor 488 and 568 (Thermo Fisher Scientific Cat#A-21202, RRID: AB-141607; #A-21206, RRID: AB-2535792; #A-10037, RRID: AB-2534013; and #A-10042, RRID: AB-2757564) were used as a secondary antibody at a 1:1000 concentration for 2 h at room temperature. Filamentous actin was labeled with phalloidin conjugated to Alexa 568 at a 1:200 concentration for 2 h.

Frozen kidney sections were permeabilized with 0.2% Triton X-100 and blocked with 5% normal goat serum. Kidney sections were fixed in 2% paraformaldehyde for 15 min and blocked with 5% normal goat serum before overnight incubation with anti-carbonic anhydrase 2 primary rabbit antibody (1:100, Abcam Cat#ab182611) in 1X PBS. Alexa Fluor 647, labeled anti-rabbit IgG, was used as a secondary antibody.

Mounting was performed using ProLong Diamond mounting medium with DAPI (Life Technologies). Images of the cochlea and kidney sections were collected at 10x-63x on Zeiss LSM 980 confocal microscope (Zeiss, Germany). ZEISS ZEN 3.3 and ImageJ [36] software were used.

## Results

### Identification of cells that are causally related to the Pendred syndrome

SV cell types were collected from WT mice to identify cells expressing the deafness-associated genes (*Slc26a4* and *Kcnj10*) of PDS and NSEVA/DFNB4. The SV was detached from the cochlea (Fig. 1A-B) to collect single cells and individual cells were harvested using the pulled-glass micropipette technique, as previously reported [26, 27]. From single cells, poly-adenylated mRNAs are reversed transcribed and amplified using Smart-Seq2 [28].

Most SV cells were round and had similar morphology, although cell size varied (Fig. 1C and S2A-D). Because of morphological similarity, we used an unbiased clustering technique to distinguish cell types. When correlated with morphology, we found that IMCs were smaller than other cell types (Fig. S2E). Specifically, the radius of IMCs (9. 52 ± 0.23 μm) was significantly smaller than the radius of SCs (10. 26 ± 0.28 μm; p = 0.043) and MCs (10. 47 ± 0.21 μm; p = 0.0033). No significant difference was found with any other pairings (Fig. S2E).

### Unbiased clustering

We completed PCA to decide which principal components (PCs) to use for clustering. The optimal number, 1–4, was determined by examining the low p-value and enrichment of genes associated with cochlear SV cells (Fig. 1D). The resultant UMAP clusters showed that cells fell into four groups (Fig. 1F). To check the quality, we examined the number of uniquely detected genes and the percentage of mitochondrial reads that map to the mitochondrial genome from each group (Fig. 1E). Low-quality dying cells have very few genes and a high percentage reads mapping to mitochondrial genes [37]. Our cell collection detected between 2,500 and 10,000 unique genes and less than 3% mitochondrial counts in most cells (Fig. 1E). These findings suggest that cell quality was excellent. Next, we quantified the uniquely expressed genes (IMC; 527, SCs; 807, RC; 414, MC; 269) and commonly expressed genes for each cell type. We observed that SC and MC have the highest similarity, with 540 genes expressed in common (Fig. 1G), likely reflecting that both MCs and SCs are anatomically marginal layer cells (Fig. 1H). In addition, we found that many genes are expressed in common in SCs and RCs, both of which express *Slc26a4*. However, perhaps surprisingly, the number of shared genes is higher between SCs and IMCs (408) than between SCs and RCs (353).

### Transcriptional profiling of cochlear SV cells

The SV is composed of three cell types based on anatomical features: (i) marginal cells (MCs), (ii) intermediate cells (IMCs), and (iii) basal cells (BCs) (Fig. 1H). The MCs are at the marginal layer of the SV, which acts as a barrier with the endolymph. The IMCs are between the MCs and BCs.

#### Known ion transporters and junctions of SV cells

IMCs are essential for maintaining endolymph homeostasis, including EP generation [38] and K^+^ recycling [15, 17, 39], functions critical for hearing. We identified in IMCs high expression of K^+^ channels (*Kcnj10*), Na^+^/K^+^-ATPase (*Atp1b3*), and gap junction 6 (*Gjb6*) genes, as has been previously reported [39–41]. These ion transporters maintain homeostasis of cochlear fluids, including endolymph and fluid in the intrastrial space (Fig. 1I). SCs highly express tight junctions (*Cldn9*, *Cldn7*, *Cldn3*) (Fig. 1I). MCs are well-known ion-transporting cells [42] that express Kcnq1/Kcne1 K^+^ channels [43], Na^+^/K^+^-ATPase (*Atp1b2*, *Atp1a1*) [44, 45], and NKCC1 co-transporters (*Slc12a2*) [46] (Fig. 1H). Our data corroborate these findings (Fig. 1I). In addition, as previously reported, pendrin encoded by *Slc26a4*, is expressed only SCs and RCs (Fig. 1H-I) [20, 24, 47–49].

#### Deafness-associated genes

To compile a list of deafness-associated genes in SV cells, we compared the transcriptome of our scRNA-seq datasets with our OtoSCOPE (otologic sequence capture of pathogenic exons) [50] database. Violin plots show gene expressions for known deafness-associated genes in SV cells (Fig. 1I). Table S1 contains a complete list of deafness-associated genes categorized by hearing loss phenotype: Online mendelian inheritance in man (OMIM) ID, cell type, and mode of inheritance. In addition, we identified potentially novel deafness-associated genes *Mpzl2* in IMCs and *Tbx1* in MCs along with the known deafness-associated genes in IMCs (*Kcnj10*, *Met*, *Mitf*, *Gjb6*, *Ednrb*, and *Gjb2* [48, 51–57]) and in MCs (*Kcnq1*, *Esrrb*, *Kcne1*, *Lrp2*, *Slc22a4*, and *Hgf* [51, 52, 58–63]).

#### Characteristics of cochlear SV cells are identified using scRNA-seq

**Fig. 2:**
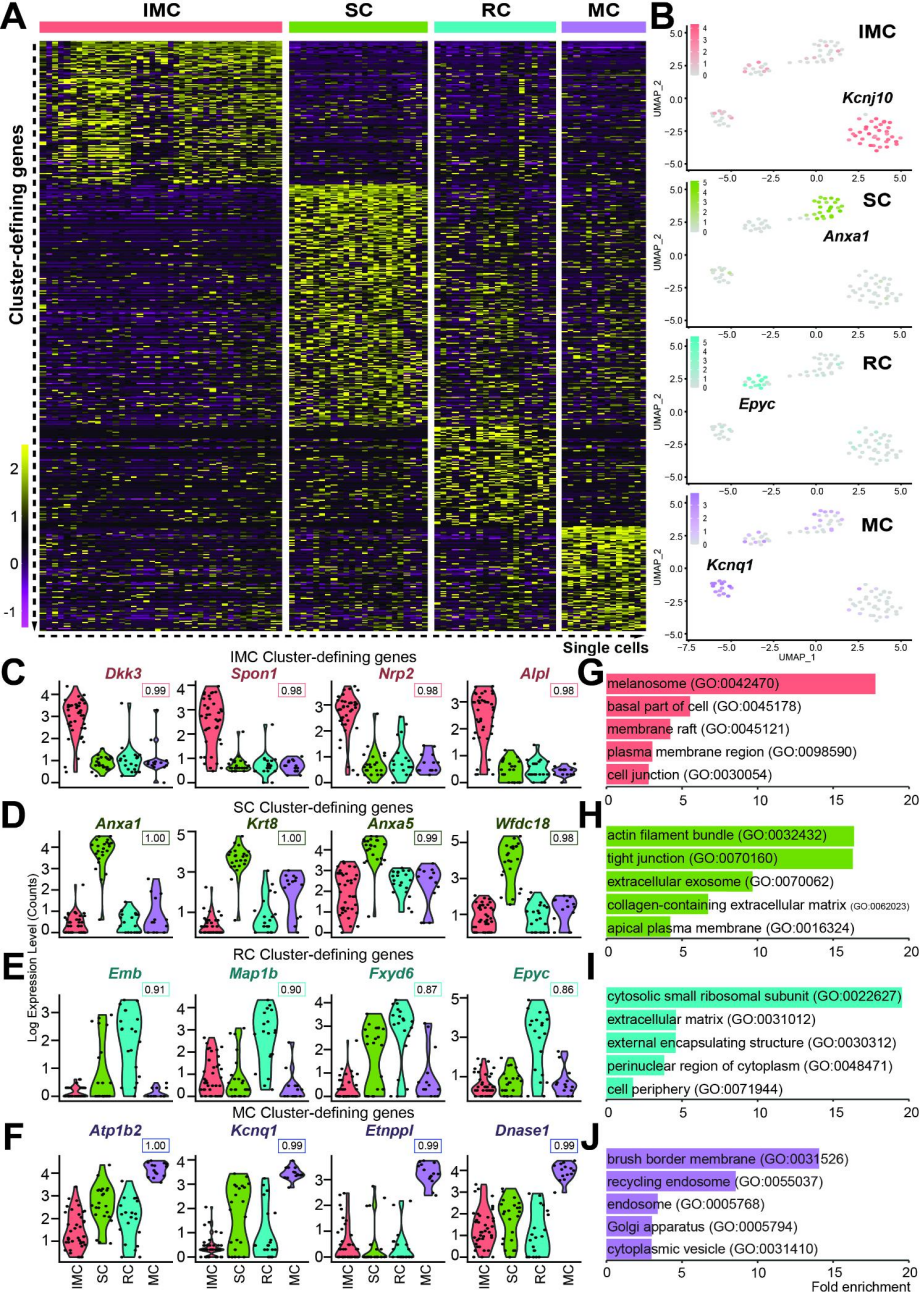
Cluster-defining genes of the stria vascularis cells, including canonical marker genes. **A**: Heatmap showing cluster-defining genes in intermediate cells (IMC), spindle cells (SC), root cells (RC), and marginal cells (MC) of the stria vascularis. Each cell group’s cluster-defining genes are ranked by ROC AUC score. **B**: The feature plot of each cell type show the expression of marker genes (IMC, *Kcnj10*; SC, *Anxa1*; RC, *Epyc*; MC, *Kcnq1*). **C-F**: Violin plots of the cluster-defining genes, including canonical marker genes. IMC (*Dkk3*, *Spon1*, *Nrp2*, *Alpl*); SC (*Anxa1*, *Krt8*, *Anxa5*, *Wdc18*); RC (*Emb*, *Map1b*, *Fxyd6*, *Epyc*); MC (*Atp1b2*, *Kcnq1*, *Etnppl*, *Dnase1*). The ROC AUC score of each gene is indicated under the name of the gene. **G-J**: Selected enriched Gene Ontology (GO) terms in the IMC (G), SC (H), RC (I), and MC (J). The melanosome, plasma membrane, membrane raft, and cell junction genes are enriched in the IMC; the SC expresses components of the extracellular exosome, the extracellular matrix is enriched in the RC, and the recycling endosome and Golgi apparatus are enriched in the MC (IMC, pink; SC, green; RC, cyan; MC, purple). The complete list of GO terms is available in supplementary tables S6-16

We used a ROC curve classifier to characterize the three distinct cell types in cochlear SV cells to find cluster-defining genes, as we have reported previously [27]. Tables S2-S5 show the cluster-defining genes for each cell type ranked by the AUC value. The heatmap of the cluster-defining genes is shown in Fig. 2A. Marker genes in SV cells (*Kcnj10* and *Nrp2* in IMC, *Axna1* in SC, *Epyc* in RC, and *Kcnq1* in MC) [51, 52] were among our cluster-defining genes (Fig. 2B-F).

We used GO-term analysis to identify the cellular components of SV cells. The cellular components of IMCs were the melanosome (GO:0042470), basal part of cell (GO:0045178), and membrane raft (GO:0045121) (Fig. 2G). MCs had intracellular components such as the brush border membrane (GO:0031526), recycling endosome (GO:0055037), endosome (GO:0005768), Golgi apparatus (GO:0005794), and cytoplasmic vesicle (GO:0031410) (Fig. 2J). In SCs and RCs, we identified components of the extracellular matrix (GO:0031012; in RCs) or extracellular exosome (GO:0070062; in SCs) (Fig. 2H-I). Exosomes are small vesicles (30–150 nm) that facilitate cell-to-cell communication by carrying cargo such as cytosolic proteins, lipids, and nucleic acids [64, 65]. Biological processes and molecular functions are listed in the supplementary tables (Tables S5-S16).

### Pendrin and other pH regulators in spindle cells of the cochlear SV

**Fig. 3:**
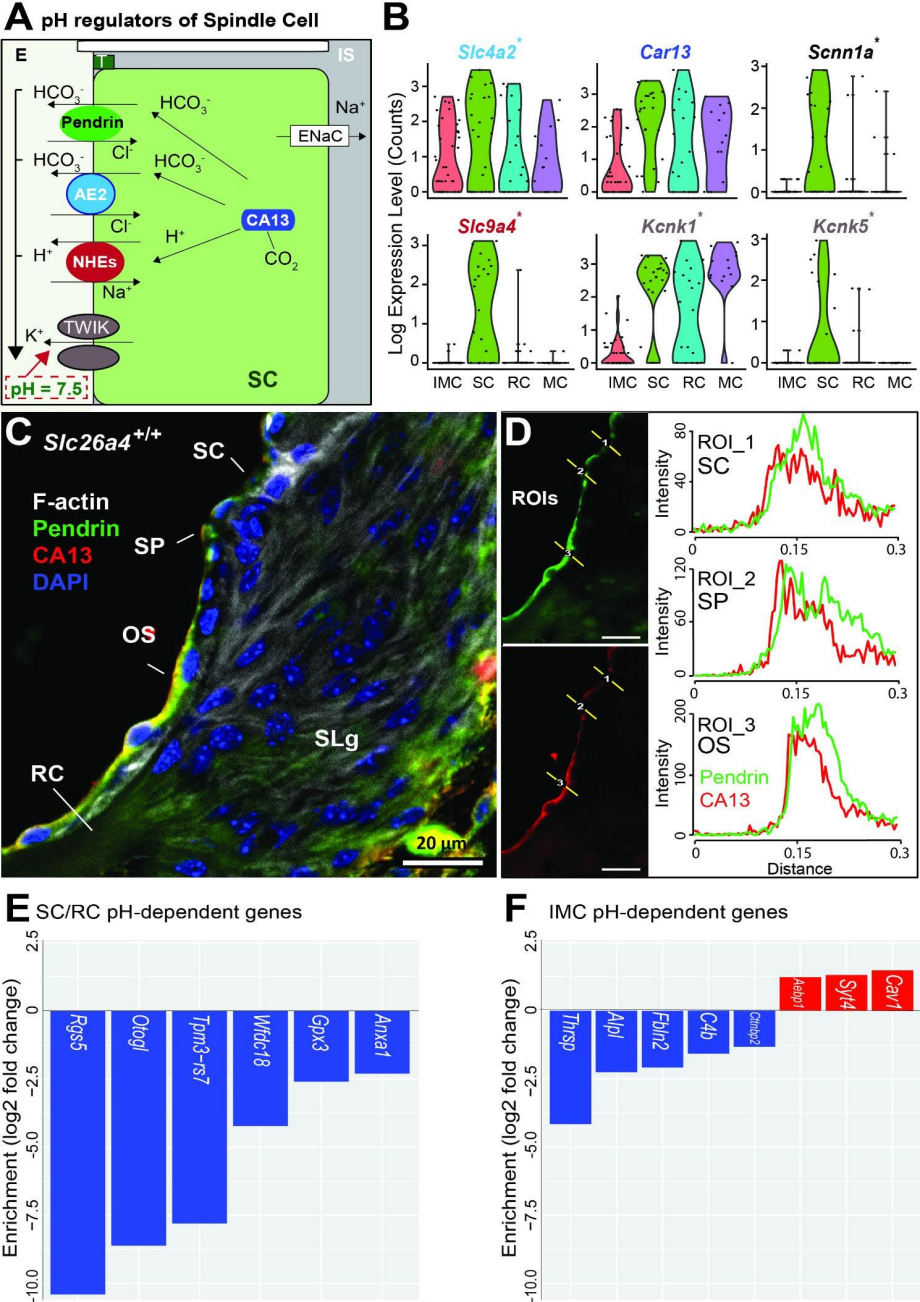
pH regulators of the spindle cell and cell-type-specific pH-dependent genes. **A**: Schematic model of pH regulation in the SC. The SC isolates the endolymph (E) from the intrastrial space (IS). Endolymph pH (7.5) is maintained by pendrin (Cl^−^/HCO_3_^−^) and by AE2, NHEs, ENac across the apical membrane of the SC, together with carbonic anhydrase (CA13) (AE2, anion exchanger 2; ENac, Epithelial Na^+^ channel; NHEs, Na^+^/H^+^ exchangers; TWIK, two-pore-domain K^+^ channel; T, tight junction). **B**: Violin plot showing the gene expression of pH regulators, including anion exchangers (*Slc4a2*), Na^+^/H^+^ exchanger (*Slc9a4*), carbonic anhydrase (*Car13*) (* indicates novel findings with pendrin expression). **C-D**: Colocalization of pendrin and intracellular carbonic anhydrase 13 in the inner ear. **C**: Representative confocal images of transversal cochlea section showing the colocalization of pendrin (green) and CA13 (red). The high magnification shows the colocalization in the SC, SP, and OS (OS, outer sulcus; RC, root cells; SC, spindle cells; SLg, spiral ligament; SP, spiral prominence; blue, nuclei; grey, F-actin). **D**: Diagram showing the line analysis (Y-axis, fluorescence intensity; X-axis, distance along the region of interest (ROI); SC; SP; OS). **E**: Bar plots of pH-dependent downregulated (blue) genes in SC/RC from *Slc26a4*^−/−^ mice compared to *Slc26a4*^+/+^ mice. Note: One gene (*Otogl*) is highly expressed in RC; the other five genes are expressed in SC. **F**: Bar plot of downregulated (blue) and upregulated (red) genes in the IMC from *Slc26a4*^−/−^ mice compared to *Slc26a4*^+/+^ mice. Note: These genes are highly expressed in IMC.

SV cells provide endolymphatic homeostasis. However, endolymph pH regulators are mainly unknown compared to ion channels involved in EP maintenance. Here, we identified the Cl^−^/HCO_3_^−^ exchanger gene (*Slc4a2*); carbonic anhydrase gene (*Car13*); Na^+^/H^+^ exchanger gene (*Slc9a4*); and epithelial Na^+^ channel (*Scnn1a*) in SCs (Fig. 3A-B). Together, these pH regulators maintain endolymphatic pH at 7.5. In addition, this specific pH also may regulate the two-pore-domain K^+^ channel (TWIK), which may be a direct source of endolymph K^+^(Fig. 3A-B). These data suggest that SCs maintain endolymph pH homeostasis using pendrin, other ion channels, and pH regulators.

#### Colocalization of pendrin and intracellular carbonic anhydrase (CAs)

We hypothesized that intracellular carbonic anhydrases (CAs), rather than membrane-bound CAs, are potential binding partners of pendrin. To confirm the co-expression of pendrin and intracellular CA at the protein level, we performed an immunostaining experiment using both cochlea and kidney and documented colocalization of pendrin and CA13 in both the cochlea (Fig. 3C-D and S4A-B) and the kidney (Fig. S4C-D).

### pH-dependent differentially expressed genes

#### Differentially expressed genes in a cell type-specific manner

We identified 121 differentially expressed genes in SCs of *Slc26a4*^+/+^ vs. *Slc26a4*^−/−^ mice (Table S17), with most genes being downregulated in the *Slc26a4*^−/−^ mouse (83% downregulated and 17% upregulated; FDR < 0.05, a mixture of a negative binomial and low-level Poisson test). There were 151 transcripts significantly altered by the loss of *Slc26a4* in IMCs (Table S18). Differential expression showed a 66% decrease and a 34% increase (FDR < 0.05, mixture of a negative binomial and low-level Poisson test) in *Slc26a4*^−/−^.

**Fig. 4:**
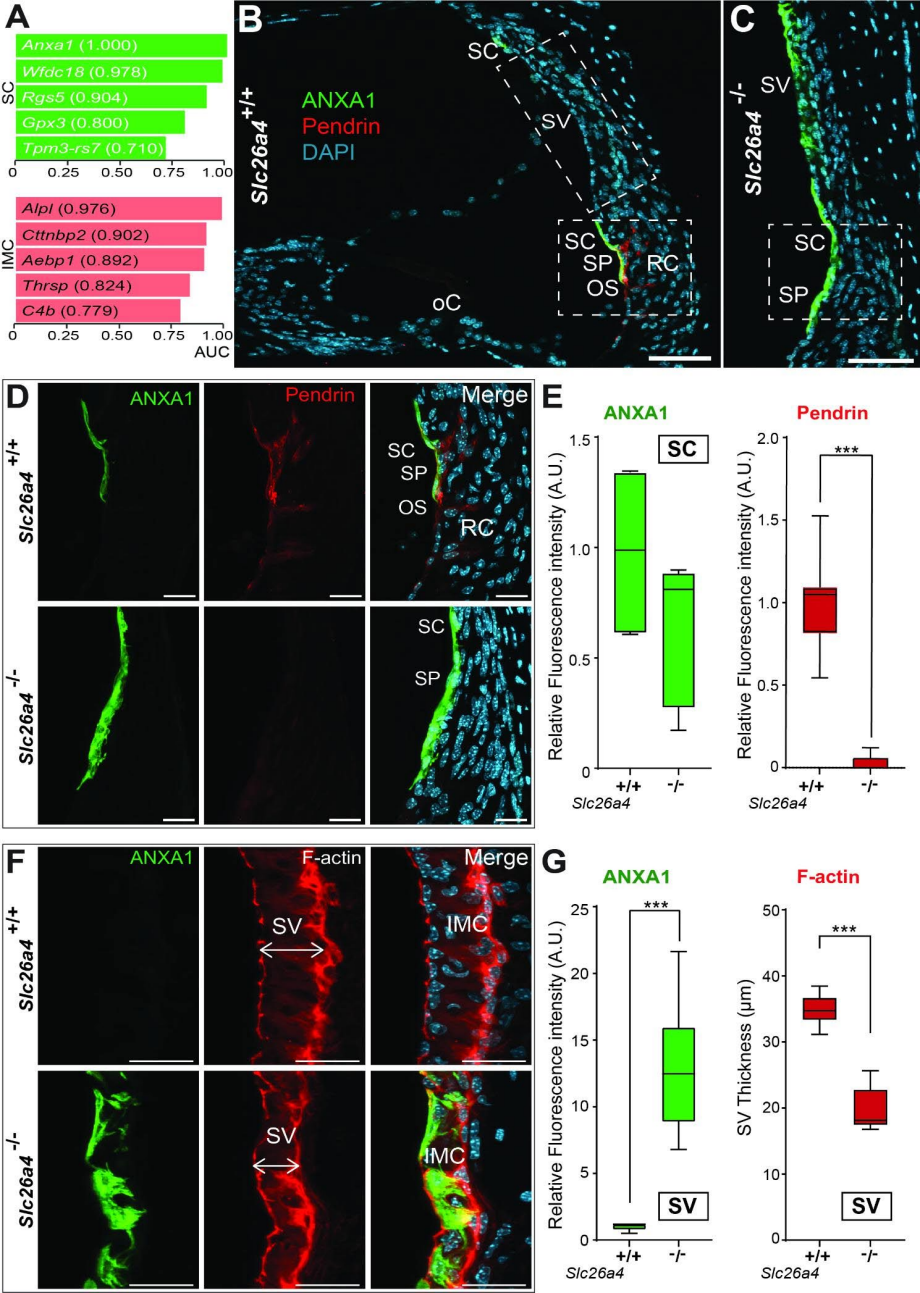
Alteration of annexin A1 localization in the stria vascularis of the *Slc26a4* mice. **A**: Rank-ordered pH-dependent genes based on ROC AUC score. **B-C**: Representative images showing cochlea section with ANXA1 (green), pendrin (red), and nuclei (cyan). Scale bar: 50 μm. (oC, organ of Corti; OS, outer sulcus; RC, root cells; SC, spindle cells; SP, spiral prominence; SV, stria vascularis). **D**: High magnification of B-C. Note: In *Slc26a4*^+/+^ mice, ANXA1 expression co-localizes with pendrin in the apical membranes of the SC. In contrast, in *Slc26a4*^−/−^ mice, without pendrin expression, the area of the ANXA1 positive cells is expanded. Scale bar: 20 μm. **E**: Box plot showing the relative fluorescence intensity of pendrin and ANXA1 in SCs. (***, p < 0.0005). In *Slc26a4*^−/−^ mice, pendrin is absent. Note: No significant difference in ANXA1 intensity was observed between *Slc26a4*^+/+^ and *Slc26a4*^−/−^ mice. **F**: High magnification of images of the SV area represented in B-C. ANXA1 (green), F-actin (red), and nuclei (cyan). SV thickness of *Slc26a4*^+/+^ and *Slc26a4*^−/−^ measured at the widest portion (site of measurement, ↔). Scale bar: 20 μm. **G**: Box plot showing the relative fluorescence intensity of ANXA1 in SV cells and SV thickness (***, p < 0.0005). Note: ANXA1 is absent in the SV of *Slc26a4*^+/+^ mice but is present in *Slc26a4*^−/−^ mice. These results may reflect the alteration of the exocytosis signaling pathway

pH-dependent genes show differences only in *Slc26a4*^+/+^ vs. *Slc26a4*^−/−^ mice (excluding differences between *Slc26a4*^+/+^ and *Slc26a4*^+/−^ mice) and included 112 genes from SCs and 133 genes from IMCs (Table S19-S20). Six pH-dependent downregulated genes (*Anxa1*, *Gpx3*, *Wfdc18*, *Tpm3-rs7*, *Otogl*, and *Rgs5*) were identified in PDCs (Fig. 3E). Five pH-dependent downregulated genes (*Cttnbp2, C4b, Fbln2, Alpl*, and *Thrsp*) and three upregulated genes (*Aebp1*, *Syt4*, and *Cav1*) were identified in IMCs (Fig. 3F). When rank-ordered by AUC score to confirm protein expression, *Anxa1* (1.000) in SCs and *Alpl* (0.976) in IMCs were ranked first for pH-dependent downregulated genes by AUC (Fig. 4A). As previously published [52], ANXA1 and pendrin are co-expressed in SCs and SP of *Slc26a4*^+/+^ mice (Fig. 4B and D). In *Slc26a4*-deficient mice, the intensity of ANXA1 was not significantly changed in SCs (Fig. 4D-E); pendrin was absent, as expected and previously reported [14, 48].

### Cellular changes lacking pendrin (***Slc26a4***)

#### Increased annexin A1 and adaptor protein 2 expression in Slc26a4^−/−^ mice

Interestingly, in *Slc26a4*^−/−^ mice, the expression of ANXA1 was found in the SC, IMCs and MCs (Fig. 4B-C F). These findings support our hypothesis that the translocation of ANXA1 from SCs to IMCs and MCs occurs through exosomes. In addition, the post-translational modification of ANXA1 allows translocation and secretion to other cell types, such as IMCs (Fig. 4B-C and F-G).

**Fig.5:**
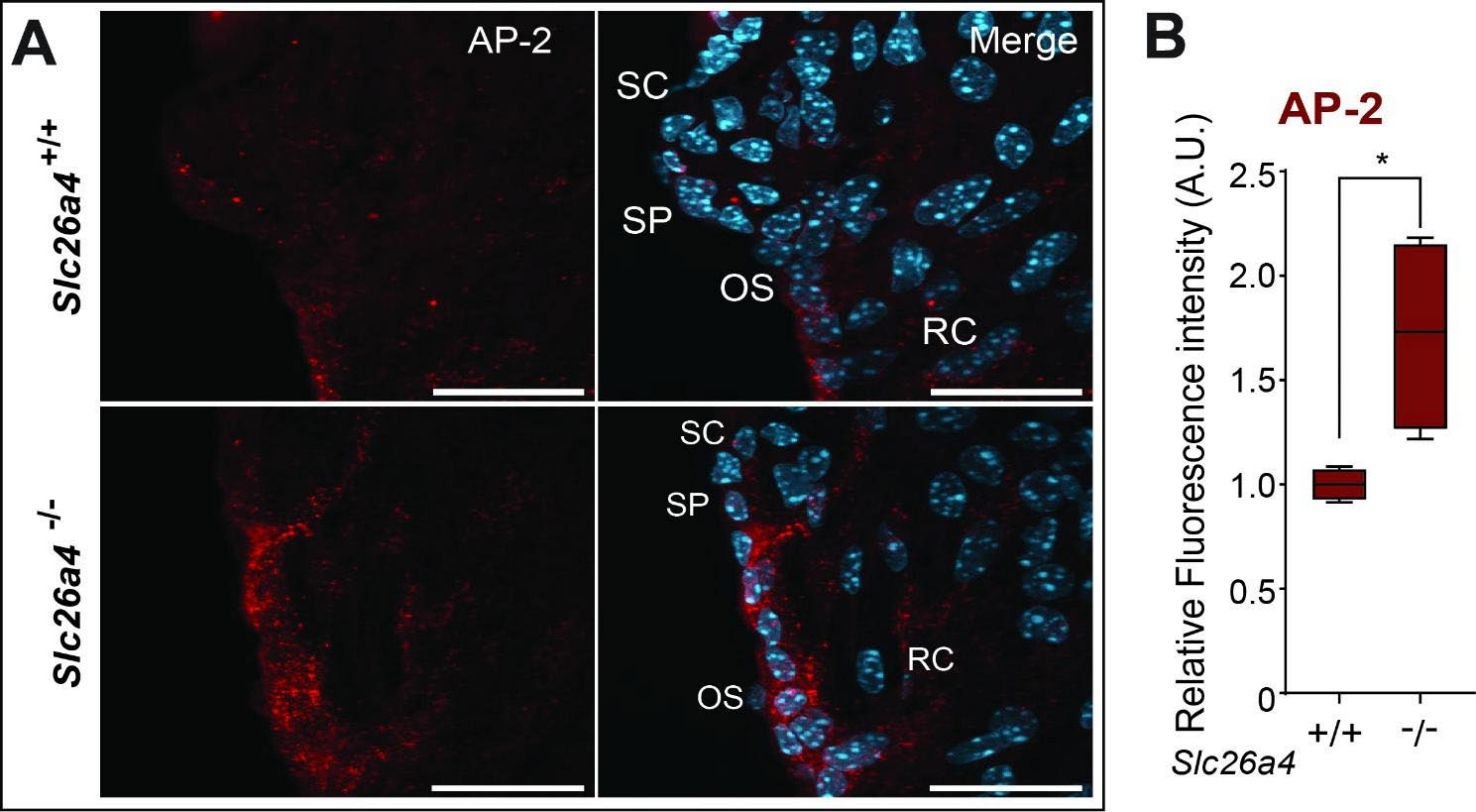
**Increased adaptor protein 2 expression in *Slc26a4***^**−/−**^**mice. A**: Representative images of the SC area showing AP-2 (red) and nuclei (cyan). Scale bar: 20 μm. (OS, outer sulcus; RC, root cells; SC, spindle cells; SP, spiral prominence). **B**: Box plot showing the relative fluorescence intensity of AP-2 in pendrin-expressing cells (*, p < 0.005). Note: In *Slc26a4*^−/−^ mice, AP-2 intensity increases in SC, SP, OS, and RC.

We hypothesized that abnormalities of PDCs and their interacting partners are the primary causes of *SLC26A4*-related hearing loss. We examined the colocalization of pendrin’s candidate binding partner to test this hypothesis. The µ2 subunit of the adaptor protein 2 (AP-2) was recently reported to bind to pendrin in the mitochondria-rich cells of the endolymphatic sac (Roux et al., 2022). AP-2 is a critical endocytic adaptor protein complex [66], which we found expressed in the PDCs of the cochlea. In addition, we showed AP-2 expression was increased in the *Slc26a4*^−/−^ mice (Fig. 5A-B).

**Fig.6:**
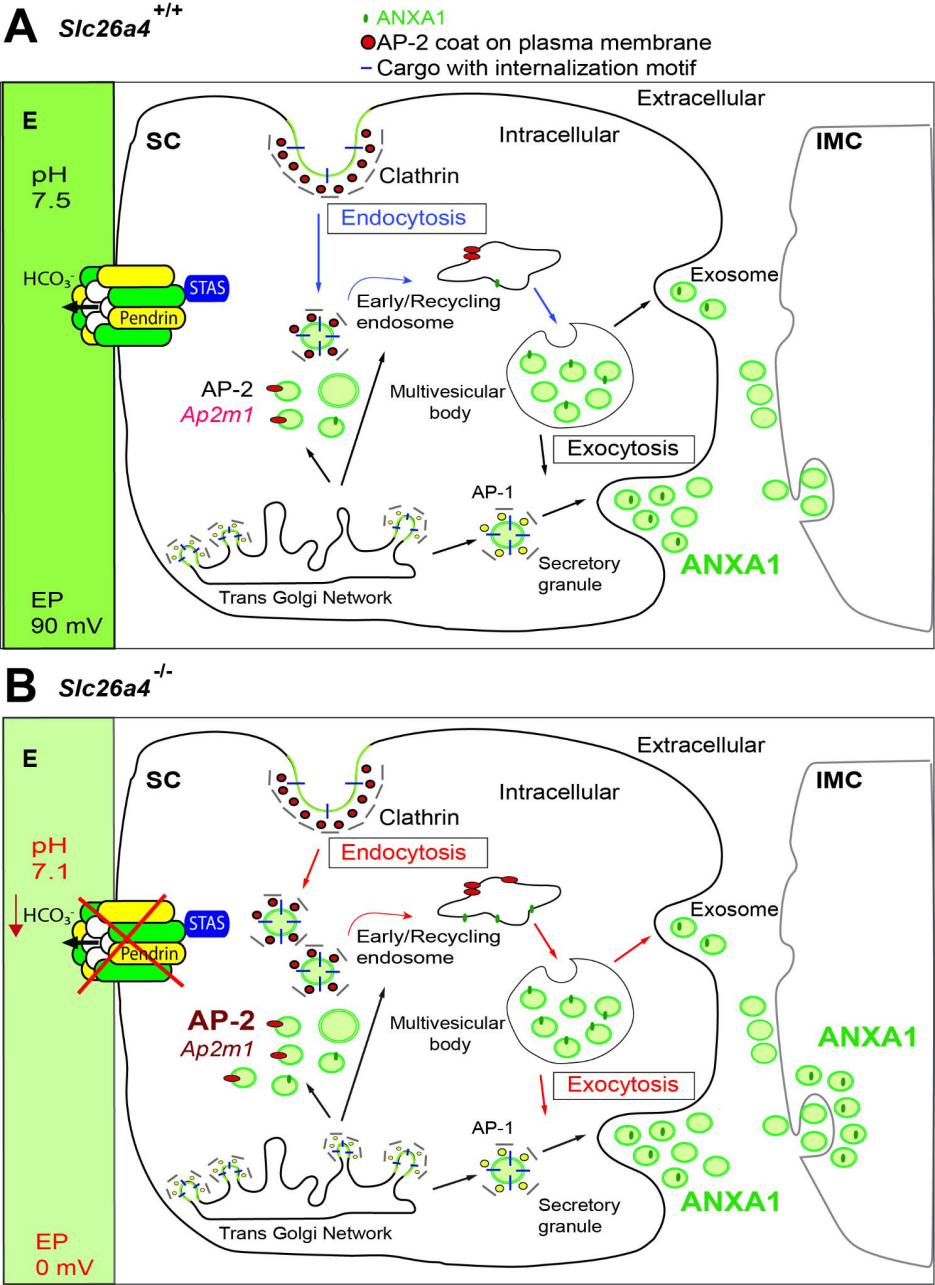
Model of exocytosis and endocytosis in the absence of pendrin. **A**: In normal conditions, pendrin transports HCO_3_^−^ into the endolymph to maintain pH homeostasis. In addition, the interaction between AP-2 and pendrin may increase the activity of pendrin in the plasma membrane and initiate clathrin-coated vesicle assembly and endocytosis. In the endocytic pathway, molecules are internalized as plasma membrane-derived clathrin-coated vesicles (blue arrows). Also, clathrin-coated vesicles carry cargo from the trans-Golgi-network and reach the plasma membrane through secretory granules (black arrows). ANXA1 in exosomes is secreted from the SCs. SCs and IMCs interact through exosomes. **B**: The interruption of the interaction between pendrin and AP-2 affects the trafficking and activity of pendrin and lowers the pH of endolymph. In addition, AP-2 increases in PDCs through increased endocytosis within the cell. In the absence of pendrin, endolymph pH drops from 7.5 to 7.1. ANXA1 in exosomes is released from the SCs and translocated and internalized in the IMCs through exocytosis (E, endolymph; EP, endolymph potential; IMC, intermediate cell; PDC, pendrin-expressing cell; SC, spindle cell)

Normally, pendrin transports HCO_3_^−^ into the endolymph to maintain endolymphatic homeostasis (EP: 90 mV and pH 7.5). Interaction between AP-2 and pendrin may increase the activity of pendrin in the plasma membrane and initiate clathrin-coated vesicle assembly and endocytosis. In the endocytic pathway, molecules are internalized as plasma membrane-derived clathrin-coated vesicles (Fig. 6A). The interruption of the interaction between pendrin and AP-2 affects the trafficking and activity of pendrin and lowers the pH of endolymph. In addition, AP-2 expression and endocytosis activity increase within the cell (Fig. 6B). In the absence of pendrin, endolymph pH drops from 7.5 to 7.1 [11, 14]. Based on this pH change (0.4 pH units) and the Henderson-Hasselbalch equation [pH = pK + log [HCO_3_^−^]/[CO_2_] (pCO_2_ = 41 mmHg, pK = 6.1)], we can calculate that endolymph HCO_3_^−^ concentration declines from 31 mM to 9.8 mM. We hypothesize that SCs and IMCs interact through exosomes and that loss of pendrin leads to an increase in the pH sensor, ANXA1, which is transported through exosomes from SCs to IMCs. In addition, the pendrin binding partner, AP-2 increased in PDCs through increased endocytosis because of the loss of the binding partner, pendrin (Fig. 6B).

## Discussion

In this study, we used scRNA-seq to investigate the gene expression profile of SV cell types to characterize cells involved in endolymphatic pH homeostasis. Our study focused on cells expressing the deafness-associated genes *Slc26a4* and *Kcnj10*. Using a micropipette-based single-cell isolation method, we selected cells with a precision that is not attainable using droplet-based systems [26, 27]. Harvesting undamaged cells during collection also facilitated the recovery of full-length transcripts, unlike the poly-A strand bias associated with other methods [67]. Limitations of the micropipette-based single-cell isolation method, however, included slow and laborious cell collection and low throughput [67]. Nevertheless, the method permitted the isolation of ultra-rare cell types that were visualized and imaged (Fig. 1C). In addition, the individual cells were of high quality, as reflected by the high number of uniquely detected genes and the low percentage of mitochondrial counts (Fig. 1E). The resulting data allowed us to identify four clusters – IMCs, SCs, RCs, and MCs – based on transcriptome profiles (Fig. 1F and 2) and provide an overall understanding of gene expression in the mature cochlear SV.

To compare gene expression profiles across IMC, SC, RC, and MC cell types, we assessed transcriptomes using a variety of approaches. Distinct differences in gene expression were recognizable on a heatmap of the cluster-defining genes in each cell type (Fig. 2A). A comparison of mean expression profiles showed that each cell type expressed unique genes (IMCs, 527; SCs, 807; RCs, 414; MCs, 269). We found that 540 genes were co-expressed only in SCs and MCs, which reflects the high transcriptome homology shared by these two cell types. As expected, both SCs and RCs show similarity, with 353 genes expressed in common in these pendrin-expressing cells. Surprisingly, we found that IMCs and SCs also had a high level of overlap (408 genes) (Fig. 1G).

Previous transcriptome profiling of SV cell types identified novel genes in a larger and more diverse cell population using the 10x Genomics Chromium platform [51, 52]. Nevertheless, despite our small sample size, we identified four cell types (IMCs, MCs, SCs, RCs) through unbiased clustering (Fig. 2B) and recapitulated known data as reported in earlier studies. In addition, we detected genes previously uncharacterized in these cell types: IMCs (*Dkk3*, *Spon1*, *Alpl*); SCs (*Krt8*, *Wfdc18*); RC (*Fxyd6*); and MCs (*Etnppl*, *Dnase1*) (Fig. 2C-F).

Endolymphatic homeostasis, including the EP, is maintained by K^+^ recycling through the SV. The K^+^ potential is generated by KCNJ10, a K^+^ channel expressed in IMCs [38]. In MCs, K^+^ is secreted into endolymph by KCNQ1/KCNE1 [40]. As might be anticipated, our data set shows that IMCs and MCs express an abundance of ion transporters (IMCs: *Kcnj10*, *Gjb6*, *Atp1b3*, MC: *Kcnq1*, *Kcne1*, *Atp1a1*, *Atp1b2*, *Slc12a2*) essential for EP generation and K^+^ recycling [15, 17, 39, 53].

Endolymph pH is maintained by PDCs (SCs and RCs) [9]. Pendrin regulates endolymph pH by transporting bicarbonate (HCO_3_^−^) across the plasma membrane (Fig. 1H-I). To identify other genes that also may play a role in maintaining endolymphatic pH, we examined the gene expression of anion exchangers, Na^+^/H^+^ exchangers, and carbonic anhydrases. We found that the Cl^−^/HCO_3_^−^ transporter gene (*Slc4a2*) and the Na^+^/H^+^ exchanger gene (*Slc9a4*) (Fig. 3A-B) are both highly expressed in SCs compared to other SV cell types. In addition, we observed a high expression of carbonic anhydrases (CAs), which are critical for pH homeostasis. CAs catalyze the reversible conversion of CO_2_ to HCO_3_^−^ (i.e., CO_2_ + CAs ↔ HCO_3_^−^ + H^+^) [18, 68, 69]. Previous studies have shown that in the pancreatic cell, the bicarbonate transporter (*Slc26a6*) binds intracellular CA (CA2) to form a transport metabolon that maximizes transport flux [68]. We hypothesized that in the cochlea, intracellular CA13 may play a similar role by functioning as an interacting partner with pendrin. Consistent with this hypothesis, we detected the expression of *Car13* (Fig. 3A-B) and confirmed the colocalization of CA13 with pendrin (Fig. 3C-D). These findings suggest that pendrin and CA13 may associate as a transport metabolon to control cochlear endolymphatic pH homeostasis.

We also detected high expression of *Kcnk1* and *Kcnk5* (Fig. 3A-B), which encode two-pore-domain K^+^ (TWIK) channels that are modulated by extracellular pH [49, 70]. Previous immunocytochemical experiments have shown that TWIK-1 (KCNK1) is highly expressed in MCs [71], while KCNK5 is mostly expressed in cochlear outer sulcus cells [49]. From these observations, we hypothesize that PDCs participate in EP regulation with known EP regulators in MCs and IMCs.

To further explore the underpinnings of endolymphatic pH regulation, we compared scRNA-seq data from the *Slc26a4*^−/−^ mouse with data from *Slc26a4*^+/−^ and *Slc26a4*^+/+^ mice. The main change associated with hearing loss in the *Slc26a4*^−/−^ mouse is acidification of the endolymphatic pH [72]. Secondary changes include loss of KCNJ10 in IMCs [48], loss of the EP [48, 72], and oxidative stress in the SV [72, 73]. Therefore, we anticipated pH-dependent alterations in the transcriptomic profiles of PDCs and IMCs in *Slc26a4*^−/−^ mice.

We completed a differentially expressed (DE) analysis of PDC and IMC to test this hypothesis. Because *Slc26a4*^+/−^ mice exhibit the same normal hearing phenotype as *Slc26a4*^+/+^ mice [13], we compared *Slc26a4*^+/+^ vs. *Slc26a4*^−/−^ mice and *Slc26a4*^+/+^ vs. *Slc26a4*^+/−^ mice to identify pH-dependent and pH-independent gene expression, respectively, in PDCs and IMCs. We reasoned that pH-dependent DE genes would be identified in *Slc26a4*^+/+^ vs. *Slc26a4*^−/−^ mice but not in *Slc26a4*^+/+^ vs. *Slc26a4*^+/−^ mice, where endolymph pH is maintained [11, 74]. These comparisons led us to identify five pH-dependent downregulated genes (*Anxa1*, *Gpx3*, *Wfdc18*, *Tpm3-rs7*, and *Rgs5*) in SCs and one pH-dependent downregulated gene (*Otogl*) in RCs (Fig. 3E). In addition, we detected five downregulated genes (*Alpl, Cttnbp2*, *Thrsp*, *C4b*, and *Fbln2*) and three upregulated genes (*Aebp1*, *Syt4*, and *Cav1*) in IMCs (Fig. 3F).

We looked in greater detail at *Anxa1* because the encoded protein, annexin A1 (ANXA1), acts as a pH and Ca^2+^ sensor and a lipid second messenger [75, 76]. It also is known to regulate cell stress with other annexins (A2 and A5) in the presence of osmotic stress due to high sodium and chloride reabsorption [75]. We found that ANXA1 is highly expressed in the apical membrane of SCs of *Slc26a4*^+/+^ mice (Fig. 2D) as previously reported [52] and colocalizes with pendrin (Fig. 4B and D). In the absence of pendrin (*Slc26a4*^−/−^ mice), ANXA1 expression expands to the SV area (IMCs and MCs) (Fig. 4C and F). We hypothesize that expanded expression reflects the activity of extracellular exosomes (Fig. 6B). The presence of extracellular exosomes in SCs but their absence in IMCs and MCs (Fig. 2G-J) suggests that abnormalities of SCs will be the primary causes of *SLC26A4*-related hearing loss. This possibility needs further validation.

The abnormality of pendrin’s interacting partner may be another cause of *SLC26A4*-related hearing loss. Thus far, the only known binding partners of pendrin are the IQ motif-containing GTPase-activating protein 1 (IQGAP1) in B-intercalated cells of the kidney [77] and AP-2 in the mitochondria-rich cells of the endolymphatic sac (Roux et al., 2022). AP-2 is critical for initiating clathrin-mediated endocytosis [78]. This study found that AP-2 was expressed only in PDCs, including SCs of the cochlea (Fig. 5A). In *Slc26a4*^−/−^ mice, AP-2 expression increased in PDCs (Fig. 5A-B). AP-2 expression may play a critical role in regulating endolymphatic pH homeostasis. Disruption of the interaction between the carboxy-terminal domain of pendrin and AP-2 may impact pendrin activity and induce pendrin endocytosis. We hypothesize that the role of AP-2 may be related to trafficking, but since other roles are possible, this function requires further investigation.

The major limitations of this study were associated with the micropipette-based single-cell isolation method, which was slow, laborious, and had low throughput [67]. In spite of these limitations, ultra-rare cell types could be isolated and were of high quality, as reflected by the high number of uniquely detected genes and the low percentage of mitochondrial counts. The resulting data offer refined insight into gene expression in the mature cochlear SV.

## Conclusions

In this study, we isolated cells expressing *Slc26a4* and *Kcnj10* from the cochlear stria vascularis; defined transcriptome profiles using scRNA-seq; identified pH-regulating genes in SCs that, together with *Slc26a4*, maintain endolymphatic pH homeostasis; and identified differentially expressed genes and proteins in a cell type-specific manner from *Slc26a4*^+/+^ and *Slc26a4*^−/−^ mice. Transcriptomic profiles of SV cells provided an understanding of pH regulator in SCs. In addition, the pH sensor, ANXA1, and the pendrin binding partner, AP-2, showed altered expression in *Slc26a4*^−/−^ mice. We propose a model in which PDCs and IMCs interact via exosomes, with increased endocytosis leading to downstream increases in AP-2 within PDCs. These findings may guide future research to clarify mechanisms essential for maintaining endolymph pH homeostasis.

## Electronic supplementary material

**Additional file 1**: **Fig. S1** Workflow. Single cells from the stria vascularis (SV) were harvested manually from P30 *Slc26a4*^+/+^, *Slc26a4*^+/−^, and *Slc26a4*^−/−^ mice as a source of full-length RNA, which was used to prepare a library for single-cell RNA sequencing. Data analysis using uniform manifold approximation and projection (UMAP) identified four types of cochlear SV cells. Cluster-defining genes from unbiased clustering analysis was used to examine gene enrichment. In addition, we used clustering-defining genes to filter each cell type. Finally, we defined pH-dependent differential expressed genes in *Slc26a4*^−/−^ as compared to *Slc26a4*^+/+^ in a cell-type- specific manner. **Fig. S2. (Related to Fig. 1)**: SV Cells and size measurement **(A-D)** Representative images of isolated cells for the four-cell type cluster. Measure the cell’s perimeter as a region of interest (ROI). Scale bar: 10 μm. (IMC, intermediate cell; MC, marginal cell; RC, root cell; SC, spindle cell). **(A)** IMCs of the cochlear SV. The median perimeter was 59.80 ± 1.42 μm (n = 30). **(B)** SCs of the cochlear SV. The median perimeter was 64.49 ± 1.73 μm (n = 19). **(C)** RCs of the cochlear SV. The median perimeter was 65.38 ± 2.34 μm (n = 11). **(D)** MCs of the cochlear SV. The median perimeter was 65.79 ± 1.29 μm (n = 16). **(E)** Box plots of the radius of each cell. Y-axis is the radius of cells *, p < 0.05. The radius of IMC (9.52 ± 0.23 μm) is significantly smaller than the radius of SC (10.26 ± 0.28 μm; p = 0.043) and MC (10.47 ± 0.21 μm; p = 0.0033). There is no difference in the radius of IMC and RC (p = 0.2). There is no difference in the radius of SC and RC (p = 0.76). There is no difference in the radius of SC and MC (p = 0. 55). There is no difference in the radius of RC and MC (p = 0.58). Each dot represents a single cell. **Fig. S3. (related to Fig. 1)** Quality control and principal components (PCs) of the SV cells. **(A)** PCs are shown as solid-colored curves. There is a sharp drop-off in p-values after the first 8 PCs. A dashed line is a uniform distribution. **(B)** A ranking of principal components (PCs) based on the percentage of variance. The black dots are 20 different PCs. **Fig. S4. (Related to Fig. 3)**: Representative confocal images showing the cellular localization of pendrin and carbonic anhydrases in the inner ear **(A-B)** and kidney **(C-D)**. **(A-B)** Immunostaining of pendrin (green) and CA13 (red) nuclei (blue), and F-actin (grey). Scale bar: 20 μm. Note: In *Slc26a4*^−/−^ mice, pendrin and CA13 staining are negative. **(C-D)** Cryosections of the mouse kidney tissue are shown as positive and negative expression controls. Scale bar: 50 μm. Note: In *Slc26a4*^+/+^ mice **(C)**, pendrin (green) and CA2 (red) co-localize in the intercalated cells. However, in *Slc26a4*^−/−^ mice **(D)**, pendrin and CA2 staining are negative

**Additional file 2**: **Table S1: OtoSCOPE v9 genes and cluster-defining genes of SV cells**. The list is ordered by the area under the ROC curve (AUC) classifier. AD: autosomal dominant, AR: autosomal recessive. IMC: intermediate cell, MC: marginal cell, RC: root cell, SC: spindle cell

**Additional file 3**: **Table S2: IMC cluster-defining genes**. The avg_diff: log Transcripts Per Million (TPM) difference in expression level between the cluster of interest and all other cells. The power means 1-β, the probability of not committing type II error. The pct.1 and pct.2 fields mean the percent of cells in which the denoted gene is detected in the IMC and other cell groups. **Table S3: SC cluster-defining genes.** The avg_diff means the logTPM difference in expression level between the cluster of interest and all other cells. The power means 1-β, the probability of not committing type II error. The pct.1 and pct.2 fields mean the percent of cells in which the denoted gene is detected in the SC and other cell groups. **Table S4: RC cluster-defining genes.** The avg_diff means the logTPM difference in expression level between the cluster of interest and all other cells. The power means 1-β, the probability of not committing type II error. The pct.1 and pct.2 fields mean the percent of cells in which the denoted gene is detected in the RC and other cell groups. **Table S5: MC cluster-defining genes.** The avg_diff means the logTPM difference in expression level between the cluster of interest and all other cells. The power means 1-β, the probability of not committing type II error. The pct.1 and pct.2 fields mean the percent of cells in which the denoted gene is detected in the MC and other cell groups. **Table S6: GO cellular component analysis of IMC, generated using PANTHER.Table S7: GO biological process analysis of IMC, generated using PANTHER.Table S8: GO molecular function analysis of IMC, generated using PANTHER.Table S9: GO cellular component analysis of SC, generated using PANTHER.Table S10: GO biological process analysis of SC, generated using PANTHER.Table S11: GO molecular function analysis of SC, generated using PANTHER.Table S12: GO cellular component analysis of RC, generated using PANTHER.Table S13: GO biological process analysis of RC, generated using PANTHER.Table S14: GO cellular component analysis of MC, generated using PANTHER.Table S15: GO biological process analysis of MC, generated using PANTHER.Table S16: GO molecular function analysis of MC, generated using PANTHER.**

**Additional file 4**: **Table S17: Differentially expressed genes in SC (*Slc26a4***^**+/+**^ vs. ***Slc26a4***^**−/−**^**).**. **Table S18: Differentially expressed genes in IMC (*Slc26a4***^**+/+**^ vs. ***Slc26a4***^**−/−**^**).**. **Table S19: Differentially expressed genes in SC (*Slc26a4***^**+/+**^ vs. ***Slc26a4***^**+/−**^**).**. **Table S20: Differentially expressed genes in IMC (*Slc26a4***^**+/+**^ vs. ***Slc26a4***^**+/−**^**).**

**Additional file 5**: R code of Seurat and SCDE

## Data Availability

The data supporting this study’s findings are currently being deposited in NCBI’s Gene Expression Omnibus to be accessible through a GEO Series accession number GSE221146 before publication. To review GEO accession GSE221146: Go to https://www.ncbi.nlm.nih.gov/geo/query/acc.cgi?acc=GSE221146.

## List of abbreviations

AE2: Anion exchanger 2
ANXA1: Annexin A1
AP-2: Adaptor protein 2
AUC: Area under the curve
BC: Basal cell
CA: Carbonic anhydrase
DE: Differentially expressed
DFNB4: Deafness, autosomal recessive 4
DPBS: Dulbecco’s phosphate-buffered saline
E: Endolymph
EP: Endocochlear potential
EVA: Enlarged vestibular aqueduct
FDR: False discovery rate
GSEA: Gene set enrichment analysis
GO: Gene ontology
IMC: Intermediate cell
IQGAP1: IQ motif-containing GTPase-activating protein 1
IS: Intrastrial space
KNN: K-nearest neighbor
MC: Marginal cell
NKCC1: Na^+^-K^+^-Cl^−^ co-transporter
NSEVA: Non-syndromic enlarged vestibular aqueduct
OS: Outer sulcus
PCA: Principal component analysis
PDC: Pendrin-expressing cell
PDS: Pendred syndrome
RC: Root cell
ROC: Receiver operating characteristic curve
SC: Spindle cell
scRNA-seq: Single-cell RNA sequencing
SCDE: Single-cell differential expression
SLg: Spiral ligament
SNHL: Sensorineural hearing loss
snRNA-seq: Single-nucleus RNA sequencing
SP: Spiral prominence
SV: Stria vascularis
UMAP: Uniform manifold approximation and projection
