# Supplementary material for: Single-cell RNA-sequencing of stria vascularis cells in the adult Slc26a4-/- mouse
Source: BMC Med Genomics. 2023 Jun 15;16:133. doi: 10.1186/s12920-023-01549-0 (PMC10268361; doi:10.1186/s12920-023-01549-0)
Supplement: Supplementary file 1 — Additional file 1: Fig. S1 Workflow. Single cells from the stria vascularis (SV) were harvested manually from P30 Slc26a4+/+, Slc26a4+/−, and Slc26a4−/− mice as a source of full-length RNA, which was used to prepare a library for single-cell RNA sequencing. Data analysis using uniform manifold approximation and projection (UMAP) identified four types of cochlear SV cells. Cluster-defining genes from unbiased clustering analysis was used to examine gene enrichment. In addition, we used clustering-defining genes to filter each cell type. Finally, we defined pH-dependent differential expressed genes in Slc26a4−/− as compared to Slc26a4+/+ in a cell-type- specific manner. Fig. S2. (Related to Fig. 1): SV Cells and size measurement (A-D) Representative images of isolated cells for the four-cell type cluster. Measure the cell’s perimeter as a region of interest (ROI). Scale bar: 10 μm. (IMC, intermediate cell; MC, marginal cell; RC, root cell; SC, spindle cell). (A) IMCs of the cochlear SV. The median perimeter was 59.80 ± 1.42 μm (n = 30). (B) SCs of the cochlear SV. The median perimeter was 64.49 ± 1.73 μm (n = 19). (C) RCs of the cochlear SV. The median perimeter was 65.38 ± 2.34 μm (n = 11). (D) MCs of the cochlear SV. The median perimeter was 65.79 ± 1.29 μm (n = 16). (E) Box plots of the radius of each cell. Y-axis is the radius of cells *, p < 0.05. The radius of IMC (9.52 ± 0.23 μm) is significantly smaller than the radius of SC (10.26 ± 0.28 μm; p = 0.043) and MC (10.47 ± 0.21 μm; p = 0.0033). There is no difference in the radius of IMC and RC (p = 0.2). There is no difference in the radius of SC and RC (p = 0.76). There is no difference in the radius of SC and MC (p = 0. 55). There is no difference in the radius of RC and MC (p = 0.58). Each dot represents a single cell. Fig. S3. (related to Fig. 1) Quality control and principal components (PCs) of the SV cells. (A) PCs are shown as solid-colored curves. There is a sharp drop-off in p-values after the first 8 PC [file 12920_2023_1549_MOESM1_ESM.pdf]

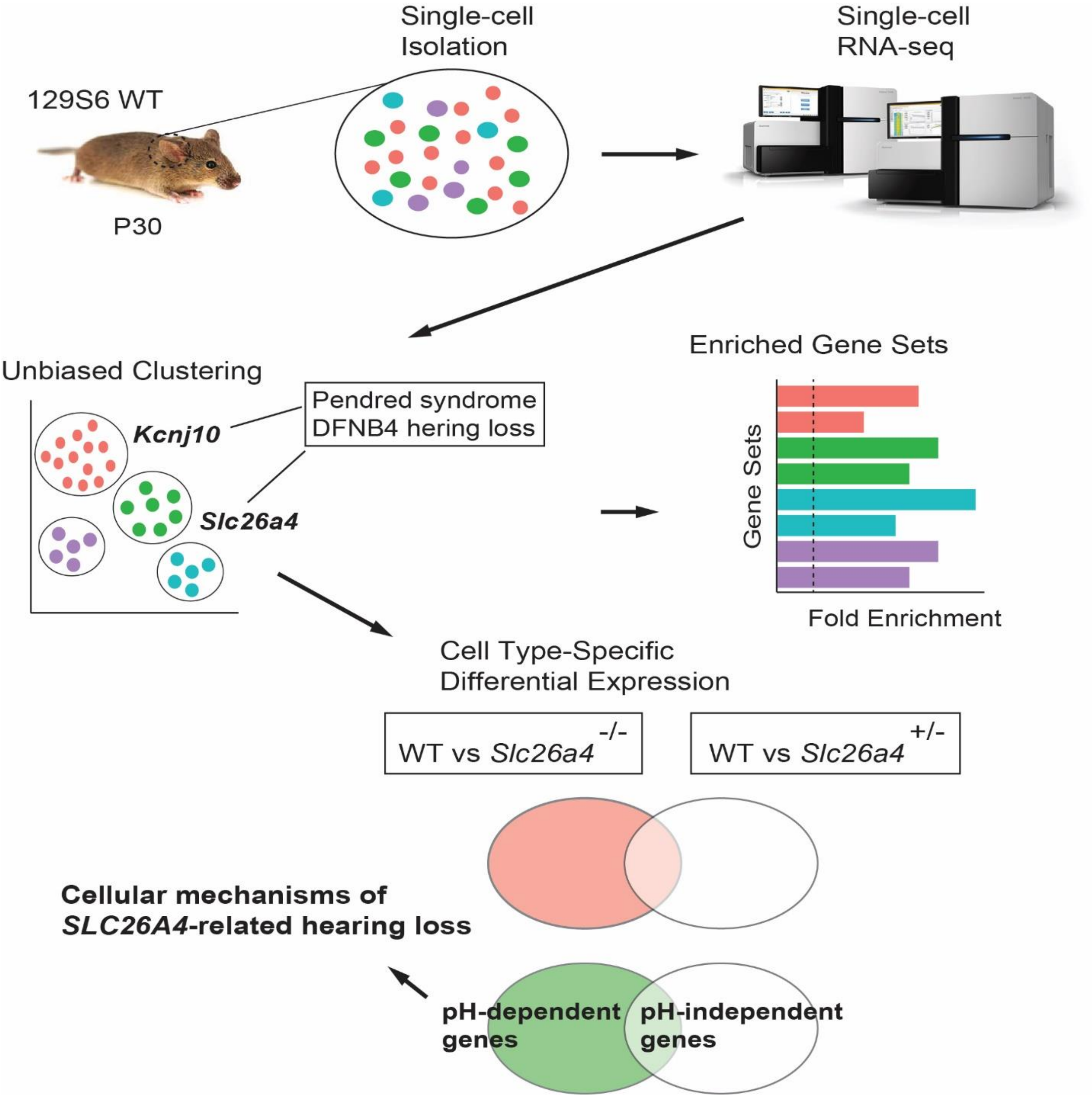

Additional file 1: Fig. S1. Workflow. Single cells from the stria vascularis (SV) were harvested manually from P30 *Slc26a4*$^{+/+}$, *Slc26a4*$^{+/-}$, and *Slc26a4*$^{-/-}$ mice as a source of full-length RNA, which was used to prepare a library for single-cell RNA sequencing. Data analysis using uniform manifold approximation and projection (UMAP) identified four types of cochlear SV cells. Cluster-defining genes from unbiased clustering analysis was used to examine gene enrichment. In addition, we used clustering-defining genes to filter each cell type. Finally, we defined pH-

dependent differential expressed genes in *Slc26a4*$^{-/-}$ as compared to *Slc26a4*$^{+/+}$ in a cell-type-specific manner.

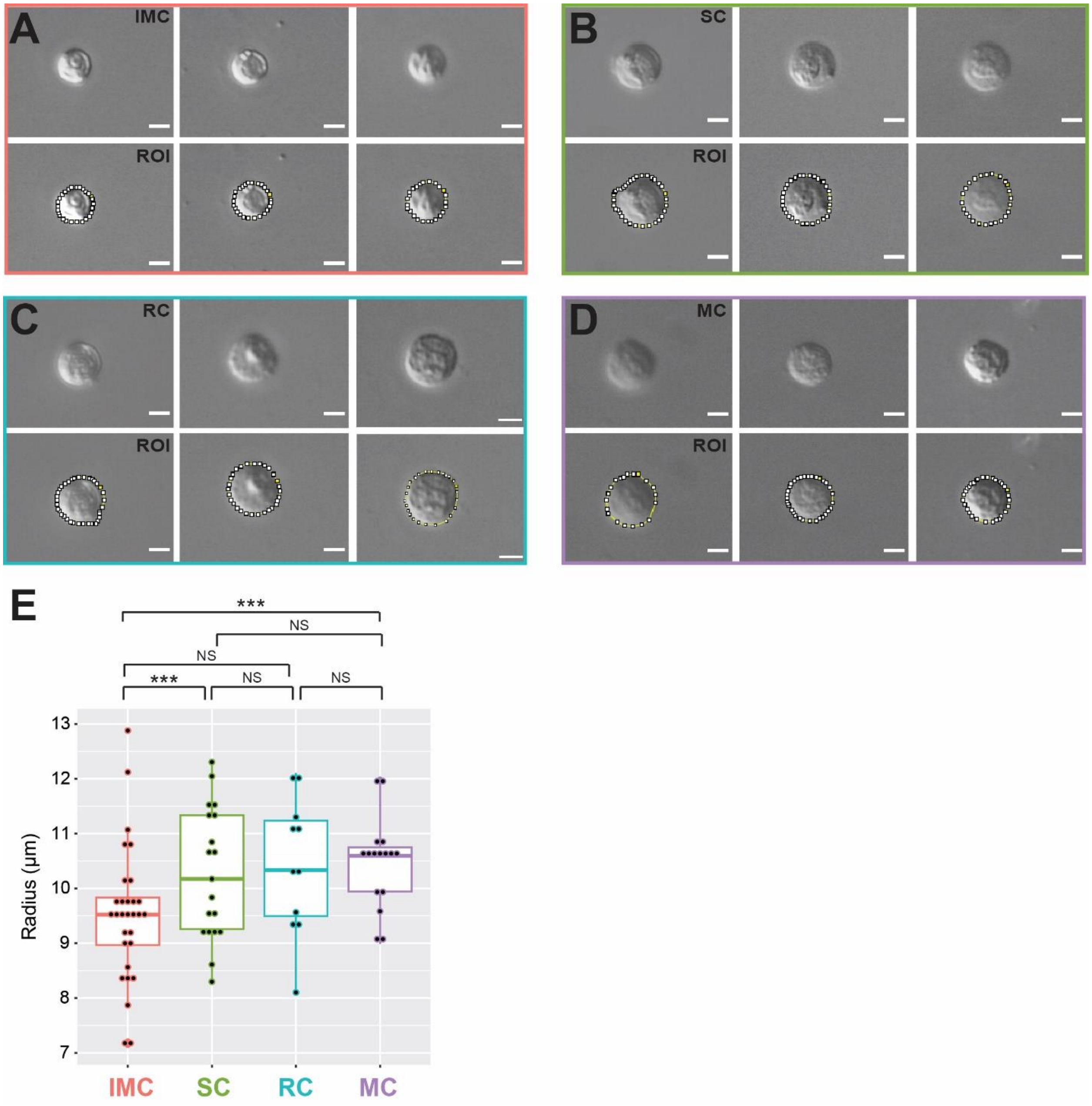

Additional file 1: Fig. S2. (Related to Figure 1): SV Cells and size measurement. **(A-D)** Representative images of isolated cells for the four-cell type cluster. Measure the cell's perimeter as a region of interest (ROI). Scale bar: 10 μm. (IMC, intermediate cell; MC, marginal cell; RC, root cell; SC, spindle cell). **(A)** IMCs of the cochlear SV. The median perimeter was 59.80 ± 1.42 μm (n=30). **(B)** SCs of the cochlear SV. The median perimeter was 64.49 ± 1.73 μm (n=19). **(C)** RCs of the cochlear SV. The median perimeter was 65.38 ± 2.34 μm (n=11). **(D)** MCs of the

cochlear SV. The median perimeter was 65.79 ± 1.29 μm (n=16). **(E)** Box plots of the radius of each cell. Y-axis is the radius of cells *, $p < 0.05$. The radius of IMC (9.52 ± 0.23 μm) is significantly smaller than the radius of SC (10.26 ± 0.28 μm; $p = 0.043$) and MC (10.47 ± 0.21 μm; $p = 0.0033$). There is no difference in the radius of IMC and RC ($p= 0.2$). There is no difference in the radius of SC and RC ($p= 0.76$). There is no difference in the radius of SC and MC ($p= 0.55$). There is no difference in the radius of RC and MC ($p= 0.58$). Each dot represents a single cell.

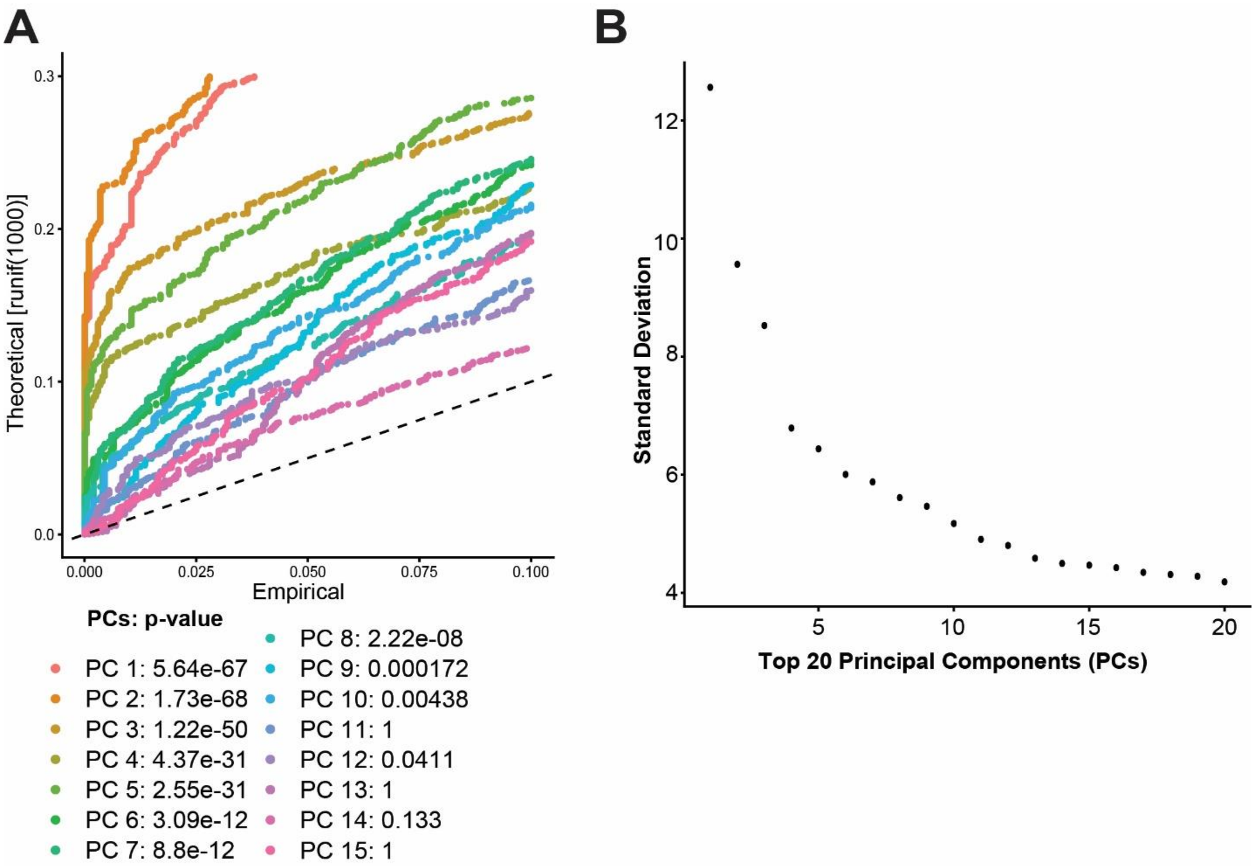

Additional file 1: Fig. S3. (Related to Figure 1): Quality control and principal components (PCs) of the SV cells. **(A)** PCs are shown as solid-colored curves. There is a sharp drop-off in p-values after the first 8 PCs. A dashed line is a uniform distribution. **(B)** A ranking of principal components (PCs) based on the percentage of variance. The black dots are 20 different PCs.

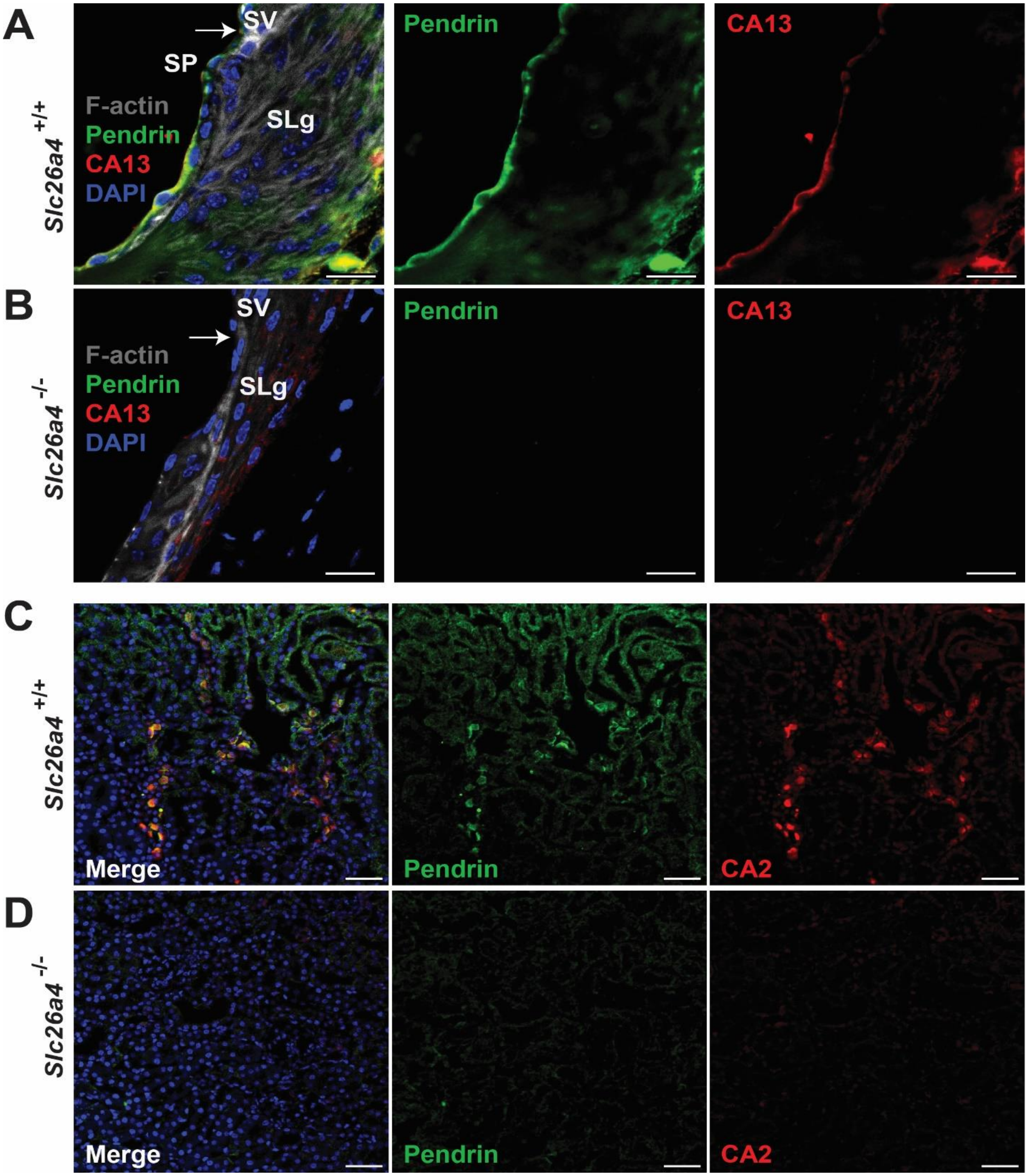

Additional file 1: Fig. S4. (Related to Figure 3): Representative confocal images showing the cellular localization of pendrin and carbonic anhydrases in the inner ear **(A-B)** and kidney **(C-D)**. **(A-B)** Immunostaining of pendrin (green) and CA13 (red) nuclei (blue), and F-actin (grey). Scale bar: 20 µm. Note: In *Slc26a4*$^{-/-}$ mice, pendrin and CA13 staining are negative. **(C-D)** Cryosections

of the mouse kidney tissue are shown as positive and negative expression controls. Scale bar: 50 μm. Note: In *Slc26a4*$^{+/+}$ mice **(C)**, pendrin (green) and CA2 (red) co-localize in the intercalated cells. However, in *Slc26a4*$^{-/-}$ mice **(D)**, pendrin and CA2 staining are negative.
