# Supplementary material for: Single-cell RNA-sequencing of stria vascularis cells in the adult Slc26a4-/- mouse
Source: BMC Med Genomics. 2023 Jun 15;16:133. doi: 10.1186/s12920-023-01549-0 (PMC10268361; doi:10.1186/s12920-023-01549-0)
Supplement: Supplementary file 5 — Additional file 5: R code of Seurat and SCDE [file 12920_2023_1549_MOESM5_ESM.pdf]

### Single Cell Differential Expression Testing

```
# Read the docs: https://satijalab.org/ (vesion 3)

# Installs:

install.packages('devtools')

install.packages('dplyr')

install.packages('Matrix')

install.packages('zoo')

install.packages('Seurat')

install.packages("tidyverse")

install.packages("dplyr")

install.packages("magrittr")

devtools::install_github("smbache/magrittr")

source("https://bioconductor.org/biocLite.R") # calls the package from the source

install.packages('BiocManager')

BiocManager::install( )

biocLite("GSEAbase")

biocLite("BiocInstaller")


library(devtools)

library(dplyr)

library(Seurat)

library(Matrix)

library(tidyverse)

library(ggplot2)

library(magrittr)


library(tximport)

library(DESeq2)

library(tidyverse)
```

```
library(GSEABase)

library(BiocParallel)

library(clusterProfiler)

library(RColorBrewer)

library(pheatmap)

library(wesanderson)

library(org.Mm.eg.db)

library(GOstats)

library(edgeR)

library(treemap)


### Load the dataset

all_cells = read.table(file.path("C:/Users/jykoh /tsv",
"renumbered_combined_transcripts_KOH_batch04.tsv"), header= TRUE, sep="\t", row.names=1)

head(all_cells)

### OHC excluding

sv_cells <- all_cells %>% select(-contains("HC"))

### Genotype selection

sv_cells_WT <- sv_cells %>% select(contains("WT"))

head(sv_cells_WT)

### Run

sv_cells_WT_SCRCMCIMC_cdSeurat <- CreateSeuratObject(counts = sv_cells_WT, project =
"sv_cells_WT_SCRCMCIMC", min.cells = 3, min.features = 200)

sv_cells_WT_SCRCMCIMC_cdSeurat

### Basic QC Stats

sv_cells_WT_SCRCMCIMC_cdSeurat[["percent.mt"]] <-
PercentageFeatureSet(sv_cells_WT_SCRCMCIMC_cdSeurat, pattern = "^mt-")

head(sv_cells_WT_SCRCMCIMC_cdSeurat@meta.data, 5)

sv_cells_WT_SCRCMCIMC_cdSeurat@meta.data

### Visualize QC metrics as a violin plot
```

```
VlnPlot(sv_cells_WT_SCRCMCIMC_cdSeurat, features = c("nFeature_RNA", "nCount_RNA", "percent.mt"), ncol = 3)

### FeatureScatter is typically used to visualize feature-feature relationships, but can be used

# for anything calculated by the object, i.e. columns in object metadata, PC scores etc.

plot1 <- FeatureScatter(sv_cells_WT_SCRCMCIMC_cdSeurat, feature1 = "nCount_RNA", feature2 = "percent.mt")

plot2 <- FeatureScatter(sv_cells_WT_SCRCMCIMC_cdSeurat, feature1 = "nCount_RNA", feature2 = "nFeature_RNA")

CombinePlots(plots = list(plot1, plot2))

### Normalizing the data

sv_cells_WT_SCRCMCIMC_cdSeurat <- NormalizeData(sv_cells_WT_SCRCMCIMC_cdSeurat, normalization.method = "LogNormalize", scale.factor = 10000)

### Detection of variable genes across the single cells

sv_cells_WT_SCRCMCIMC_cdSeurat <- FindVariableFeatures(sv_cells_WT_SCRCMCIMC_cdSeurat, selection.method = "vst", nfeatures = 2000)

### Identify the 10 most highly variable genes

top10 <- head(VariableFeatures(sv_cells_WT_SCRCMCIMC_cdSeurat), 10)

### plot variable features with and without labels

plot1 <- VariableFeaturePlot(sv_cells_WT_SCRCMCIMC_cdSeurat)

plot1

plot2 <- LabelPoints(plot = plot1, points = top10, repel = TRUE, xnudge = 0, ynudge = 0)

plot2

### Scaling the data and removing unwanted sources of variation

all.genes <- rownames(sv_cells_WT_SCRCMCIMC_cdSeurat)

sv_cells_WT_SCRCMCIMC_cdSeurat <- ScaleData(sv_cells_WT_SCRCMCIMC_cdSeurat, features = all.genes)

### Perform linear dimensional reduction

sv_cells_WT_SCRCMCIMC_cdSeurat <- RunPCA(sv_cells_WT_SCRCMCIMC_cdSeurat,  features = VariableFeatures(object = sv_cells_WT_SCRCMCIMC_cdSeurat))

### Examine and visualize PCA results a few different ways

print(sv_cells_WT_SCRCMCIMC_cdSeurat[["pca"]], dims = 1:5, nfeatures = 5)
```

```
VizDimLoadings(sv_cells_WT_SCRCMCIMC_cdSeurat, dims = 1:2, reduction = "pca")

VizDimLoadings(sv_cells_WT_SCRCMCIMC_cdSeurat, dims = 3:4, reduction = "pca")

DimHeatmap(sv_cells_WT_SCRCMCIMC_cdSeurat, dims = 1:4, cells = 97, balanced = TRUE)

### Determine the 'dimensionality' of the dataset

# NOTE: This process can take a long time for big datasets, comment out for expediency. More

# approximate techniques such as those implemented in ElbowPlot() can be used to reduce computation time

sv_cells_WT_SCRCMCIMC_cdSeurat <- JackStraw(sv_cells_WT_SCRCMCIMC_cdSeurat, num.replicate = 100)

sv_cells_WT_SCRCMCIMC_cdSeurat <- ScoreJackStraw(sv_cells_WT_SCRCMCIMC_cdSeurat, dims = 1:20)

JackStrawPlot(sv_cells_WT_SCRCMCIMC_cdSeurat, dims = 1:15)

ElbowPlot(sv_cells_WT_SCRCMCIMC_cdSeurat)

### Determine statistically significant principal components

# NOTE: This process can take a long time for big datasets, comment out for expediency.

### Cluster the cells

sv_cells_WT_SCRCMCIMC_cdSeurat <- FindNeighbors(sv_cells_WT_SCRCMCIMC_cdSeurat, dims = 1:4)

sv_cells_WT_SCRCMCIMC_cdSeurat <- FindClusters(sv_cells_WT_SCRCMCIMC_cdSeurat, resolution = 0.8)

head(Idents(sv_cells_WT_SCRCMCIMC_cdSeurat), 5)

### Run non-linear dimensional reduction (UMAP/tSNE)

# If you haven't installed UMAP, you can do so via reticulate::py_install(packages = 'umap-learn')

#library(reticulate)

sv_cells_WT_SCRCMCIMC_cdSeurat <- RunUMAP(sv_cells_WT_SCRCMCIMC_cdSeurat, dims = 1:4)

### find all markers of cluster 0

cluster0.markers <- FindMarkers(sv_cells_WT_SCRCMCIMC_cdSeurat, ident.1 = 0, min.pct = 0.25)

head(cluster0.markers, n = 50)

### find all markers of cluster 1

cluster1.markers <- FindMarkers(sv_cells_WT_SCRCMCIMC_cdSeurat, ident.1 = 1, min.pct = 0.25)

head(cluster1.markers, n = 50)

### find all markers of cluster 2
```

cluster2.markers <- FindMarkers(sv_cells_WT_SCRCMCIMC_cdSeurat, ident.1 = 2, min.pct = 0.25)

head(cluster2.markers, n = 50)

cluster3.markers <- FindMarkers(sv_cells_WT_SCRCMCIMC_cdSeurat, ident.1 = 3, min.pct = 0.25)

head(cluster3.markers, n = 50)

### find markers for every cluster compared to all remaining cells, report only the positive ones

sv_cells_WT_SCRCMCIMC_cdSeurat.markers <- FindAllMarkers(sv_cells_WT_SCRCMCIMC_cdSeurat, only.pos = TRUE, min.pct = 0.25, logfc.threshold = 0.25)

### ROC

cluster0.markers <- FindMarkers(sv_cells_WT_SCRCMCIMC_cdSeurat, ident.1 = 0, logfc.threshold = 0.25, test.use = "roc", only.pos = TRUE)

cluster1.markers <- FindMarkers(sv_cells_WT_SCRCMCIMC_cdSeurat, ident.1 = 1, logfc.threshold = 0.25, test.use = "roc", only.pos = TRUE)

cluster2.markers <- FindMarkers(sv_cells_WT_SCRCMCIMC_cdSeurat, ident.1 = 2, logfc.threshold = 0.25, test.use = "roc", only.pos = TRUE)

cluster3.markers <- FindMarkers(sv_cells_WT_SCRCMCIMC_cdSeurat, ident.1 = 3, logfc.threshold = 0.25, test.use = "roc", only.pos = TRUE)

head(cluster0.markers, n = 5)

head(cluster1.markers, n = 5)

head(cluster2.markers, n = 5)

head(cluster3.markers, n = 5)

### canonical marker genes of sv

FeaturePlot(sv_cells_WT_SCRCMCIMC_cdSeurat, features = c("Kcnj10"), pt.size = 2, cols = c("lightgrey", "#F8766D"))

FeaturePlot(sv_cells_WT_SCRCMCIMC_cdSeurat, features = c("Anxa1"), pt.size = 2, cols = c("lightgrey", "#7CAE00"))

FeaturePlot(sv_cells_WT_SCRCMCIMC_cdSeurat, features = c("Epyc"), pt.size = 2, cols = c("lightgrey", "#00BFC4"))

FeaturePlot(sv_cells_WT_SCRCMCIMC_cdSeurat, features = c("Kcnq1"), pt.size = 2, cols = c("lightgrey", "#C77CFF"))

VlnPlot(sv_cells_WT_SCRCMCIMC_cdSeurat, features = c("Kcnj10", "Anxa1", "Epyc", "Kcnq1", "Slc26a4"), slot = "counts", log = TRUE)

### Heatmap

```
clusterdefining <- sv_cells_WT_SCRCMCIMC_cdSeurat.markers %>% group_by(cluster)

DoHeatmap(sv_cells_WT_SCRCMCIMC_cdSeurat, features = clusterdefining$gene)


### SCDE

### Preparing data for SC (PDC)

PDC <- read.table(file.path("C:/Users/Jin-Young/OneDrive - University of Iowa/tsv", "PDCs_Geno_ID.txt"),
header= TRUE, sep="\t", row.names=1)

PDC_m <- as.matrix(PDC)

### Pre_factor determining

#PDCs

y_WTKO <- gsub("^PDC(.*)_.*", "\\1", colnames(WTKO_PDC_m))

l2cols_y_WTKO <- c("coral4", "slateblue3")[as.integer(factor(y_WTKO, levels = c("WT","KO")))]

y_WTHE <- gsub("^PDC(.*)_.*", "\\1", colnames(WTHE_PDC_m))

l2cols_y_WTHE <- c("coral4", "slateblue3")[as.integer(factor(y_WTHE, levels = c("WT","HE")))]

### Factor determining cell types

#For PDC WT and KO cells

sg_PDC_WT_KO <- factor(gsub("(PDCWT|PDCKO).*", "\\1", colnames(WTKO_PDC)), levels=c("PDCWT",
"PDCKO"))

sg_PDC_WT_HE <- factor(gsub("(PDCWT|PDCHE).*", "\\1", colnames(WTHE_PDC)), levels=c("PDCWT",
"PDCHE"))

### The group factor should be named accordingly

# sg_PDC

names(sg_PDC_WT_KO) <- colnames(sg_PDC_WT_KO)

table(sg_PDC_WT_KO) # PDCWT: 51 , PDCKO: 25

names(sg_PDC_WT_HE) <- colnames(sg_PDC_WT_HE)

table(sg_PDC_WT_HE) # PDCWT: 51 , PDCHE: 13

### Fitting error models

# takes too long to run on travis…

library('scde')
```

WTKO_PDC_knn <- knn.error.models(WTKO_PDC_m, k = ncol(WTKO_PDC_m)/2, n.cores = 4, min.count.threshold = 2, min.nonfailed = 5, max.model.plots = 10)

#this step takes a considerable amount of time (~14 min) unless multiple cores are used. I'm using 4 cores

head(WTKO_PDC_knn)

# filter out cells that don’t show positive correlation with the expected expression magnitudes (very poor fits)

valid.cells_WTKO_PDC <- WTKO_PDC_knn$corr.a > 0

table(valid.cells_WTKO_PDC) # valid.cells_PDC: TRUE: 76

WTKO_PDC_knn <- WTKO_PDC_knn[valid.cells_WTKO_PDC, ]

# get number of genes per cell

genes.per.cell_WTKO_PDC <- apply(WTKO_PDC_m, 2, function(x) sum(x > 10))

hist(genes.per.cell_WTKO_PDC)

# estimate gene expression prior

o.prior_WTKO_PDC <- scde.expression.prior(models = WTKO_PDC_knn, counts = WTKO_PDC_m, length.out=400, show.plot=T)

# Testing for differential expression

groups4 <- factor(gsub("(PDCWT|PDCKO).*", "\\1", rownames(WTKO_PDC_knn)), levels=c("PDCWT", "PDCKO"))

groups5 <- factor(gsub("(PDCWT|PDCHE).*", "\\1", rownames(WTHE_PDC_knn)), levels=c("PDCWT", "PDCHE"))

ediff_WTKO_PDC <- scde.expression.difference(WTKO_PDC_knn, WTKO_PDC_m, o.prior_WTKO_PDC, groups = groups4, n.randomizations=100, n.cores=4, verbose=1) ## takes time

# top regulated genes

head(ediff_WTKO_PDC[order(ediff_WTKO_PDC$Z, decreasing  =  TRUE), ])

p.values_WTKO_PDC <- 2*pnorm(abs(ediff_WTKO_PDC$Z),lower.tail=F) # 2-tailed p-value

p.values.adj_WTKO_PDC <- 2*pnorm(abs(ediff_WTKO_PDC$cZ),lower.tail=F) # Adjusted to control for FDR

significant.genes_WTKO_PDC<- which(p.values.adj_WTKO_PDC<0.05)

length(significant.genes_WTKO_PDC)

# significantly diff expressed genes after multiple testing correction

ord_WTKO_PDC <- order(p.values.adj_WTKO_PDC[significant.genes_WTKO_PDC]) # order by p-value

```
de_WTKO_PDC <- cbind(ediff_WTKO_PDC[significant.genes_WTKO_PDC,1:3],
p.values.adj_WTKO_PDC[significant.genes_WTKO_PDC])[ord_WTKO_PDC,]

colnames(de_WTKO_PDC) <- c("Lower bound","log2 fold change","Upper bound","p-value")
```
